# Integrated Circuits on Fiber Substrates: State-of-the-Art System-on-Fiber Technologies for Smart Textiles and Wearables

**DOI:** 10.1007/s40820-025-02056-w

**Published:** 2026-02-03

**Authors:** Juyoung Jin, Jonghyun Won, Daegun Kim, Shiva Kumar Arumugasamy, Sungjun Park, Tae-Wook Kim

**Affiliations:** 1 Department of Flexible and Printable Electronics, LANL‐JBNU Engineering Institute‐Korea, Jeonbuk National University, Jeonju, 54896 Republic of Korea; 2 Department of Intelligence Semiconductor Engineering, Ajou University, Suwon, 16499 Republic of Korea; 3 School of Chemical, Biological and Battery Engineering, Gachon University, Seongnam, 13120 Republic of Korea; 4 Department of Electrical and Computer Engineering, Ajou University, Suwon, 16499 Republic of Korea; 5 Department of JBNU-KIST Industry-Academia Convergence Research, Jeonbuk National University, Jeonju, 54896 Republic of Korea

**Keywords:** Electronic fiber, E-textile, Integration, Multifunctional e-textile, Wearable electronics

## Abstract

System-on-fiber technologies have emerged as a promising platform for seamless integration sensing, signal processing, and communication functionalities within textile-compatible fiber architectures. Advances in materials science and microscale fabrication have enabled the development of multifunctional fibers that serve as active components in large-scale woven systems. These fibers can perform a range of functions including sensing, data processing, and even neuromorphic computing. Despite their potential applications in wearable electronics, healthcare monitoring, and human–machine interfaces, the practical implementation stays in its infancy. Key challenges include limitation in device encapsulation, interconnect reliability, and scalable manufacturing. This review systematically summarizes recent advancements in manufacturing approaches for fiber-based integrated electronics, device configurations, and integration strategies. Furthermore, key technological hurdles and future opportunities for achieving fully integrated autonomous fiber-based electronic systems are discussed.

## Highlights

- Presents a hierarchical overview of system-on-fiber (SoF) technologies, linking materials, fabrication methods, and device architectures from single-fiber electronics to system-level intelligent textiles.
- Establishes a quantitative process–performance correlation framework, integrating AI-driven material optimization and comparative metrics (e.g., yield, endurance, and conductivity retention) across coating, thermal drawing, deposition, and spinning techniques.
- Proposes a standardization and industrial translation roadmap outlining key steps testing certification, scalable manufacturing, and modular integration to move SoF systems from laboratory prototypes to consumer-ready smart textiles.

## Graphical abstract

**Figure:**
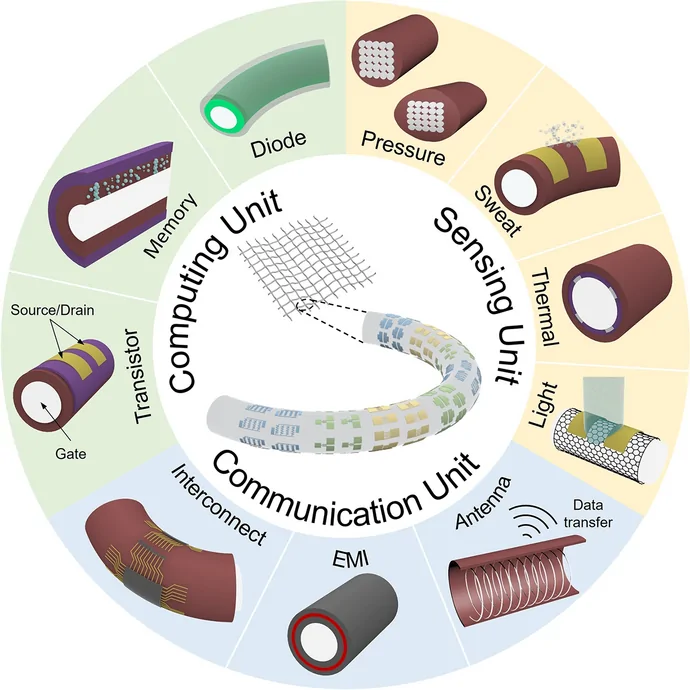

## Introduction

The rapid growth of smart factories, widespread 5G adoption, and emerging technologies like autonomous vehicles, artificial intelligence (AI), and the Internet of Things (IoT) are transforming industries. This evolution increases demand for highly integrated, compact, energy-efficient, and reliable electronic systems [1]. Innovations such as very-large-scale integration (VLSI), biomedical sensors, system-on-chip (SoC), microelectromechanical systems (MEMS), tissue-on-a-Chip, Monolithic three-dimensional (3D) integrated circuits (ICs), and high-bandwidth memory (HBM) are crucial for enhancing system performance and versatility. Additionally, neural recording technologies and advanced computing paradigms are essential for tackling contemporary technological and societal challenges [2].

Fiber-shaped electronic devices are gaining attention for their ability to enable complex integration in constrained environments, a challenge for conventional planar electronic device architectures [3]. The devices significantly reduce the system size by fabricating ICs on one-dimensional (1D) substrates, achieving high integration densities through advanced manufacturing techniques. Additionally, they can be seamlessly integrated into large-area textiles using established production methods like weaving or braiding, easing the development of wearable systems for continuous health monitoring, environmental sensing, and communication [4].

**Fig. 1:**
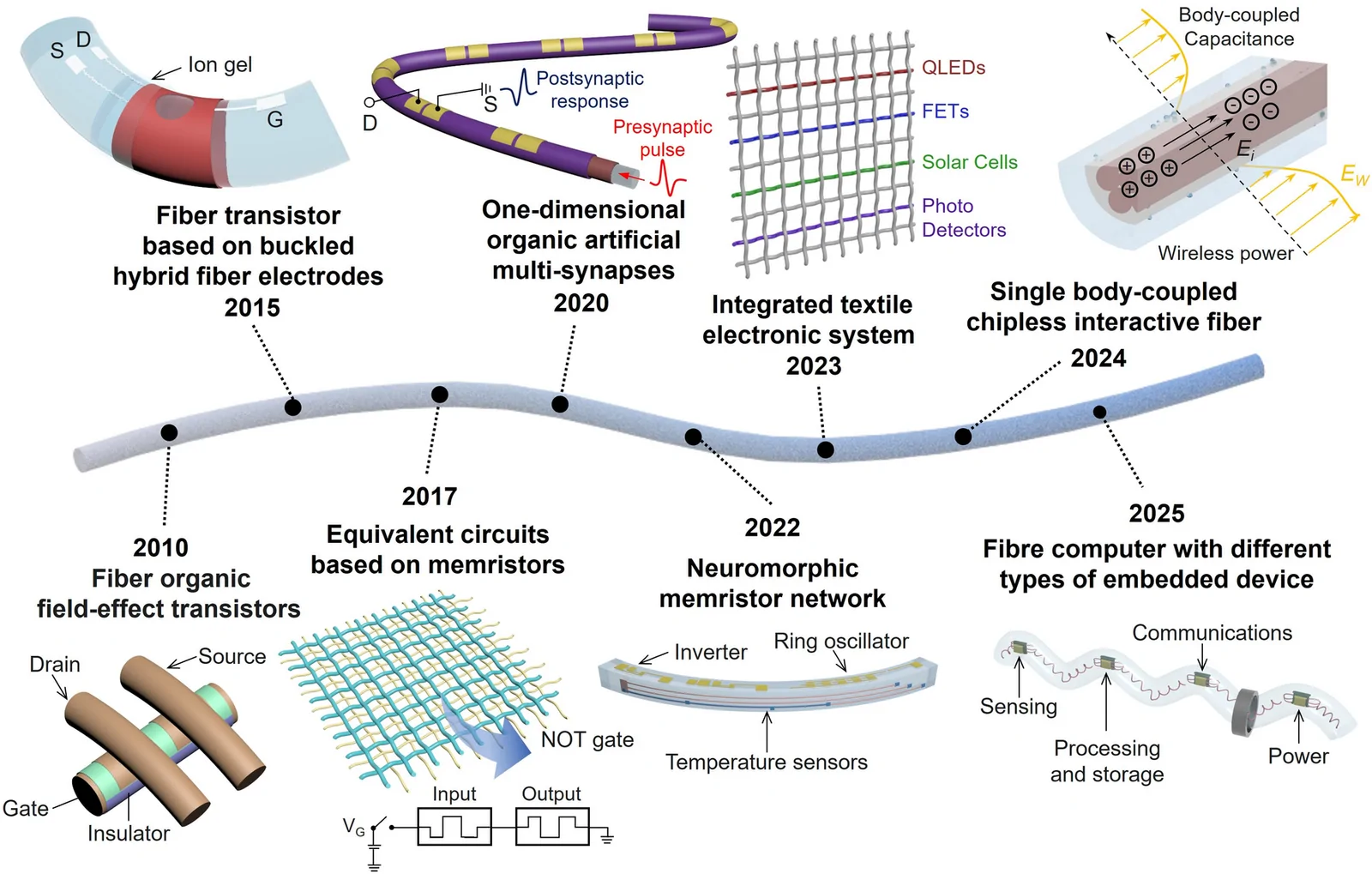
Timeline of platform technology development for smart textiles and wearable electronics

Fabrics offer flexible and multifunctional surfaces ideal for integrated electronic systems [5]. As the wearable electronics market expands, fiber-based technologies spanning 1D and two-dimensional (2D) showcase advantages like breathability, mechanical flexibility, and unobtrusive integration into daily life [6]. This review collectively addresses these technologies as fiber-based electronic devices with 1D and 2D configurations. Figure 1 illustrates a comprehensive timeline outlining the significant milestones in the development of fiber-based electronics, particularly emphasizing their progression toward fully integrated textile electronic systems incorporating integrated circuit (IC) technology.

Fiber-based electronic technologies aim to emulate the seamless and highly efficient integration of biological systems. Such systems inherently coordinate diverse functions including energy harvesting, storage, context-aware sensing, and data communication. Achieving this level of integration and multifunctionality in fiber-based electronics is the next frontier of wearable technology. However, challenges such as complete circuit-level integration onto fibers, reliable data acquisition, secure handling of personal information, and on-fiber computing remain largely underexplored, offering significant opportunities for future research and development [4, 7].

Unlike planar microelectronic platforms, System-on-Fiber (SoF) architectures exploit the 1D geometry and mechanical compliance of fibers to achieve seamless integration within textiles, enabling next-generation applications in wearable healthcare, soft robotics, environmental monitoring, and human–machine interfaces. By embedding active materials and circuit components within or along the fiber core, SoF systems can perform signal processing, data transmission, and energy harvesting directly at the textile level, bridging the gap between microelectronics and macroscopic fabrics. Recent advancements in fabrication techniques have laid the groundwork for scalable, kilometer-long multifunctional fibers that keep both mechanical flexibility and electrical stability under repeated deformation [3, 8]. However, translating these laboratory-scale prototypes into integrated, autonomous, and intellectual fiber networks requires breakthroughs in materials optimization, architectural design, and AI-driven manufacturing control. Establishing a unified roadmap that couples material innovation with system-level co-integration and adaptive data intelligence will be pivotal for fully self-controlled, self-learning textile systems marking a fundamental step toward the future of AI-enhanced electronic fibers.

SoF technologies have emerged as a transformative direction in wearable electronics, enabled by the convergence of flexible electronics, advanced fiber engineering, and multifunctional materials. Unlike conventional e-textiles that attach rigid components or printed conductors onto fabrics, SoF architecture integrates sensing, logic, memory, power, and wireless functions directly into 1D fibers, offering superior mechanical compliance, lightweight operation, and long-term wearability. Recent advances in multi-material thermal drawing, nanoscale deposition, coaxial patterning, liquid–metal encapsulation, and high-resolution transfer printing demonstrate that fibers can serve both as passive carriers and as active computing nodes, memory elements, micro-energy units, and antennas. Despite these breakthroughs, current progress remains fragmented, with limited system-level coordination, interconnect reliability challenges, and insufficient quantitative links between fabrication conditions and device performance. This review therefore provides a unified system-level perspective on SoF technologies, covering materials, device modules, integrated fiber circuits, and textile-scale systems, while highlighting emerging directions such as AI-assisted materials optimization, digital-twin manufacturing, and adaptive fiber networks.

Recent advancements have led to the development of various fiber-based energy conversion and storage devices, sensors, and actuators, to understand the emergent field with excellent reviews have been published on these specific components [9, 10]. However, the existing literature focuses on individual device types and their performance metrics, lacking a comprehensive review of fiber-based electronic devices within fully integrated smart systems. This review addresses this gap by providing an overview of the recent developments in fiber-shaped electronic devices and their integration into intelligent systems. The review is organized as follows: Sect. 2 outlines the manufacturing techniques and production processes for integrated electronic fibers. Section 3 discusses the operational principles of the computing, sensing, and communication units that are essential components of these systems. Section 4 explores the device architecture on 1D substrates. Finally, Sect. 5 shows the key challenges and unresolved issues hindering the advancement of fiber-shaped electronic devices and innovative fiber technologies.

## Fabrication of 1D Electronic Fibers

Recent advancements in fiber electronics focus on developing flexible and stretchable electronic devices and their components, such as functional elements, advanced materials, and compliant substrates [8, 11, 12]. Unlike conventional electronics on rigid 2D substrates, fiber electronics are based on 1D platforms, presenting unique design and manufacturing challenges. This requires scalable fabrication techniques like melt printing, electrospinning, electrodeposition, chemical vapor deposition (CVD), casting, rolling, molding, and thermal drawing [13, 14]. These techniques improve the precise patterning and uniform deposition of active materials along the fiber axis, enhancing functionality and performance. Moreover, the shape, composition, and internal architecture of electronic fibers can be finely engineered by selecting suitable soft materials and tailoring fabrication strategies to enhance their functionality and performance [15, 16]. Furthermore, Table 1 depicts a summary of the fabrication techniques, along with their key features, and device effects.

**Table 1:** A summary of the fabrication techniques, along with their key features, and device effects

Method	Role	Salient features	Limitations	Industrial adaptability	Device/system effects	Applicability
Coating	Deposition of functional/conductive layers on fiber surfaces	Low-cost, scalable, compatible with roll-to-roll production Tunable thickness Post-functionalization	Uniform wetting and adhesion on porous textiles can be difficult Limited control on nanoscale roughness	High	Enhanced carrier mobility, reduced contact resistance, improved bending stability	Conductive coatings, encapsulation, sensing and barrier layers
Thermal drawing	Co-integration of multiple materials (metal, polymer, semiconductor) in a continuous fiber	Precise internal architecture Kilometer-scale scalability Optical-fiber infrastructure ready	Limited to thermally compatible materials Viscosity mismatch issues	High	Stable conductivity under strain, improved optical/electrical uniformity in long fibers	Integrated electrodes, optical/electronic fibers, system-on-fiber cores
Deposition	Formation of thin, conformal functional films	Atomic-level thickness control High uniformity and interfacial quality	High vacuum cost Limited flexibility for thick coatings	Moderate	Controlled threshold voltage, reduced leakage current, improved device lifetime	Dielectrics, semiconductor films, encapsulation, barrier coatings
Patterning	Define circuit layouts and micro/nano features	High spatial partible Compatible with digital design Reconfigurable	Complex alignment on curved fibers Limited resolution for soft substrates	Moderate	High logic fidelity, minimized signal crosstalk, scalable circuit integration	Microelectrodes, interconnects, sensor arrays, photonic circuits
Spinning	Fiber formation and material blending	Continuous fiber production Excellent material diversity Tunable morphology	Electrospinning has low throughput Wet spinning limits minimum diameter	High	Tunable capacitance and tensile strength, enhanced sensitivity for strain/pressure sensors	Conductive fibers, stretchable sensors, energy storage, textiles

Improvements in optical fiber manufacturing have further advanced fiber-based electronic devices, easing microscale material structuring in continuous fiber formats [17]. Integrating multiple electronic components into fiber or textiles demands meticulously designed architectures and fabrication strategies to ensure seamless operation, durability, and robust electrical interconnectivity. Traditional textile techniques such as weaving and braiding are now effective for large-area production and increased integration density. These advances pave the way for developing next-generation electronic textiles (e-textiles) with multifunctional capabilities.

Fabrication strategies for producing standalone functional fibers can be broadly classified into three main categories: spinning, thermal drawing, and printing techniques [18]. Other methods, including fibrillation, extrusion, in situ growth, electrochemical coating, the pad-dry-cure method, and spray coating, have also been explored [19–22]. Coating, thermal drawing, deposition, and spinning are the most widely adopted owing to their versatility and compatibility with various materials and device architectures.

### Solution-Based Surface Coating Techniques

**Fig. 2:**
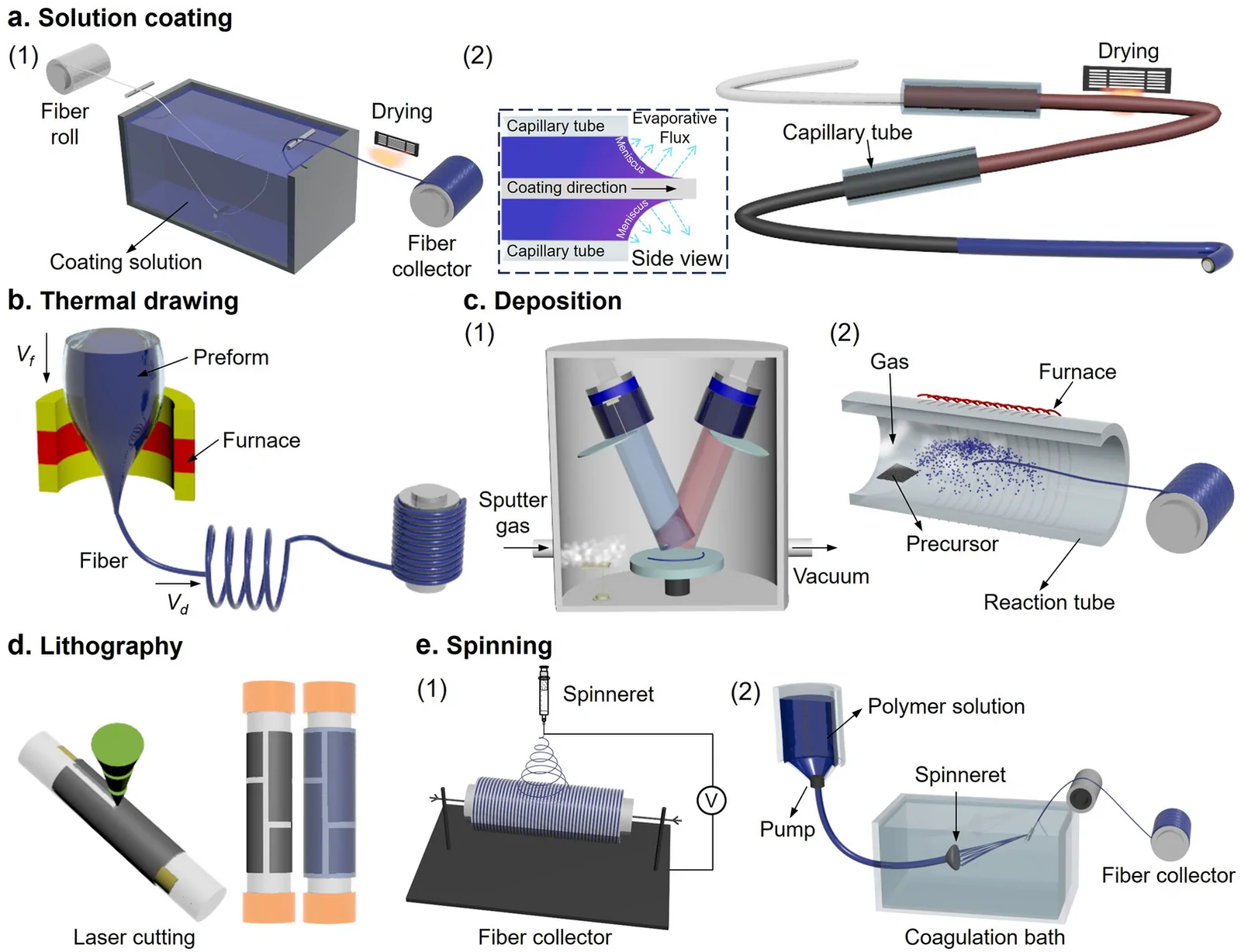
Fabrication techniques illustration. **a** Solution coating (1) Schematic of the set-up used to produce continuous fiber electronics. (2) Schematic representation of a continuous reel-to-reel coating process for fabrication of the fiber organic memory, along with an illustration and photograph of a P(VDF-TrFE)-wrapped metal wire formed by the CTAC process. **b** Thermal drawing: Illustration of the fiber being drawn through the drawing process. **c** Deposition (1) Schematic of the magnetron sputtering modification process. (2) CVD method. **d** Laser patterning: Schematic showing the laser-based fabrication process and its electrode structure. **e** Spinning (1) Schematic of basic equipment for electrospinning. (2) Schematic illustration of the wet-spinning process

Surface coating of fibers is a key fabrication strategy that involves depositing functional material layers onto fiber surfaces to enhance intrinsic properties or introduce new functionalities [23]. Coatings can be applied using various solution-based methods, including dip coating and wrapping coating, as depicted in Fig. 2a. These techniques provide efficient material usage, scalability, and precise control over film morphology. Coating processes, including reel-to-reel dip-coating, slot-die, and inkjet printing are particularly favorable for industrial implementation due to their compatibility with continuous and roll-to-roll textile production lines, enabling scalable deposition of conductive, semiconductive, or encapsulating layers with precise control over thickness and morphology [24]. However, successful coating requires careful consideration of the wetting behavior of the fiber substrate with the solution. To address this challenge, studies have focused on improving the uniformity and adhesion of coatings on various fiber materials [25].

#### Dip-Coating Methods for Conformal Film Deposition

Solution coating processes typically involve depositing a functional film onto the outer surface of the fiber core using methods such as dip coating or solvent evaporation [26]. In dip coating, the fiber is immersed in a coating solution and slowly withdrawn, allowing the solvent to evaporate and form a thin, uniform solid layer (Fig. 2a(1)) [27]. This technique is favored for its simplicity and the ability to coat curved surfaces conformally. Hwang et al*.* developed a fiber-based white organic light-emitting diode (WOLED) using a single emission layer fabricated by dip coating [28]. This method effectively addressed geometry and structural challenges by conformally coating the fiber, while the device was encapsulated with an Al_2_O_3_/elastomer bilayer to enhance its durability against sweat and external mechanical stimuli. WOLED demonstrated stable operation in saline solution for over 300 min without dark spots and supported its performance under 100 load cycles (~ 1 N) with minimal degradation. Zhai et al*.* fabricated viologen-based electrochromic fibers using a continuous layer-by-layer coating approach by incorporating a transparent conductive network of Au-coated Ag nanowires into a parallel dual-electrode configuration [29]. The electrochemical stability of the conductive layer was significantly improved by epitaxial Au shell growth on the Ag nanowires, suppressing oxidation and improving long-term durability.

#### Capillary Tube-Assisted Coating (CTAC)

In solution-based approaches, fibers are passed through narrow tubes to enhance coating uniformity and control film thickness. Despite challenges like nonuniform film formation (e.g., the “pearl necklace” effect from capillary instabilities) and weak adhesion, this technique remains appealing owing to its simplicity, low material consumption, and continuous processability [30]. Kang et al*.* showed a fiber-based organic transistor memory using a CTAC system (Fig. 2a(2)) [31]. This method enabled the formation of smooth, compact nanogranular P(VDF-TrFE) films on flexible metal wires. Adjusting coating speed and solution concentration minimizes material waste while ensuring high-quality deposition.

### Thermal Drawing of Multicomponent Fibers

Thermal drawing is a fiber fabrication technique that involves heating a macroscopic preform composed of multiple functional components and later elongating it into a thin, flexible fiber, while preserving the internal architecture of the original structure [32, 33]. Thermal drawing is one of the most industrially mature and scalable approaches, capable of producing kilometer-long multifunctional fibers with integrated electrodes, insulators, and semiconductors in a single step. Its compatibility with existing optical-fiber manufacturing infrastructure ensures reproducibility, mechanical robustness, and cost efficiency at large volumes [34]. This top-down approach enables embedding complex electronic, optical, and sensing elements within the fiber, allowing elevated levels of integration and scalability.

This technique integrates multiple electronic components into a single fiber and was first proven by Fink et al. in a thermally drawn fiber, leading to multifunctional optoelectronic fibers [35–37]. Functional chips are interconnected with conductive wires within a polymeric preform, which is thermally drawn into a single fiber, while keeping the relative positions and electrical connectivity of the embedded components. This approach simplifies the assembly of fiber-based systems with integrated sensing, energy, or computational functions.

Thermal drawing enables the creation of intricate internal architectures within the fiber cross section, unlike traditional weaving methods [38, 39]. However, its high-temperature requirement limits its use with thermally unstable materials. Additionally, undesired chemical interactions between heterogeneous materials can compromise device functionality. Therefore, carefully selecting compatible materials and perform designs is essential to support device integrity.

The continuous fabrication of kilometer-long fibers with complex, aligned internal structures is achieved by arranging components, such as metal electrodes and semiconductor cores, within a macroscopic preform. Heating the performance above the glass transition temperature (*T*_g_) of the cladding material and drawing it at a controlled speed preserves the relative positions of the internal components (Fig. 2b) [40]. This technique has been successfully applied to various materials, including polyvinylidene fluoride (PVDF) and PVDF combined with barium titanate, used as piezoelectric and triboelectric layers in energy-harvesting devices. The thermal, mechanical, and chemical properties of the materials must be carefully matched to prevent deformation, delamination, and chemical reactions during drawing.

Compared with other fiber fabrication techniques, thermal drawing offers notable advantages in scalability, mechanical robustness, electrical stability, and precise control over internal architecture. Embedding electronic components within a continuous polymer matrix enhances wearable applications by providing washing resistance and structural stability under deformation [41]. This technique allows spatially precise placement of functional materials, enabling controlled electrode spacing and adjustable semiconductor layer thickness [34, 42]. However, thermal drawing requires specialized equipment, and careful material choice is crucial to avoid thermal degradation and undesired chemical reactions. The surrounding polymer matrix may limit light transmission to embedded optoelectronic components, reducing efficiency. Additionally, integrating nanomaterials into electrically connected architecture stays challenging due to the mismatched properties of the constituent materials. Addressing these limitations is essential for advancing high-performance fiber-integrated electronic systems.

### Deposition Process for Functional Thin Films

Depositing thin films onto fibers or fiber-shaped substrates is crucial for integrating functional layers, such as electrodes, semiconductors, dielectrics, and protective coatings. Vapor-phase methods, including physical vapor deposition (PVD), CVD, and atomic layer deposition (ALD) are widely used owing to their ability to create uniform, conformal films formation on curved and nonplanar geometries, essential for the high-performance fiber-based electronic systems. Furthermore, in deposition methods such as PVD, CVD, and ALD are critical for forming high-quality thin films and conformal interfaces; their adaptability to automated and spatial-ALD systems allows mass production with atomic precision, supporting the fabrication of encapsulation layers, dielectric coatings, and high-performance semiconducting shells.

PVD converts solid materials into vapor that condenses on substrates making it a standard method for depositing electrode using metal masks or patterned templates. Variants include thermal evaporation, electron-beam evaporation, and magnetron sputtering [43–45]. Sputtering offers precise control over process parameters such as gas flow rate, gas composition, target material, and applied power. This enables tunability for exact adjustment of the film thickness and composition, particularly oxygen content in the metal oxides as illustrated in (Fig. 2c(1)). This makes PVD suitable for engineering oxide-based electronic devices and integrating electrodes into cylindrical fiber substrates. Optimized sputtering conditions enable fine control over the oxygen ratios in conductive oxides, improving conductivity and durability [46].

CVD is a bottom-up approach that grows nanomaterials such as particles, nanotubes, wires, 2D materials, graphene, and metal–organic frameworks [47–50] by introducing gaseous precursors into a chamber where they react or decompose on a heated substrate (Fig. 2c(2)). Plasma-enhanced CVD improves film quality by lowering the required reaction temperature. For instance, Zeng et al. showed the vertical growth of 3D graphene sheets on polyacrylonitrile fibers using thermal CVD, resulting in superior electrical conductivity and electromagnetic shielding compared to conventional graphene materials [51]. This study highlights the potential of CVD for producing functional architectures directly on fiber substrates.

ALD employs sequential exposure of the substrate to two or more precursors, allowing for one molecular layer per cycle. This results in precise control over film thickness and excellent step coverage, enabling conformality on complex surfaces that is challenging to achieve with conventional methods [52]. ALD is particularly valuable for forming dielectric, oxide, or encapsulation layers on fiber substrates, making it essential for advanced device integration in nonplanar and flexible structures. Although ALD provides unmatched conformality and atomic-scale thickness control, its scalability and manufacturability remain significant challenges for large-scale fiber production. The inherently slow deposition rate (< 1 nm min^−1^) and high vacuum-system cost restrict throughput and make continuous coating economically demanding [53, 54]. Moreover, ALD films such as Al_2_O_3_ or HfO_2_ can suffer from hydrolytic degradation, cracking, or delamination under repeated bending or washing, limiting long-term durability in textile environments. Recent advances such as hybrid encapsulation (ALD + elastomer layers), spatial ALD, and roll-to-roll configurations show promise in improving both mechanical stability and process speed, achieving > 90% barrier retention after 1,000 cycles and deposition rates approaching 1 m min^−1^. Looking ahead, the integration of AI-assisted process control and digital-twin modeling will be vital for optimizing precursor flow, temperature gradients, and cycle timing, enabling ALD to evolve from a laboratory precision method into a cost-efficient, high-throughput encapsulation strategy suitable for industrial SoF engineering [55, 56].

Successful implementation of these thin-film deposition techniques requires ensuring compatibility between process conditions and the physical and chemical properties of fibers. Key factors include processing temperature, plasma energy, and chemical reactivity of the precursor gases, all of which must align with the thermal stability, mechanical integrity, and chemical resistance of the chosen fiber material. Selecting fibers with suitable chemical durability is crucial to achieving uniform film formation while supporting the structural and functional integrity of the device.

### Patterning Process on Fiber Substrates

Patterning techniques are crucial for integrating functional electronic components into fiber substrates with a high spatial resolution. Traditional lithography involves coating a substrate with a photoresist, exposing it to ultraviolet light through a patterned mask, etching the exposed areas, and removing the photoresist to reveal the desired structure. Methods including electron-beam lithography, focused ion beam milling, direct laser writing, projection photolithography, and interference patterning have been developed. Therefore, patterning techniques including laser ablation, photolithography, and inkjet/screen printing enable precise circuit definition and device miniaturization, and are well-suited for digitally reconfigurable, automated processing in large-scale textile integration. However, adapting these processes to curved or cylindrical fibers poses unique challenges [57–60].

To address this, a rotating patterning approach was introduced, wherein fiber rotates while scanned by a linear ultraviolet light source to enable uniform exposure on its curved surface. Despite this, device coverage stays limited owing to the need for precise synchronization between fiber rotation and light scanning. Modified lithographic methods have been applied to profiled fibers, such as square or strip geometries, where conventional planar patterning tools can be more effective. These advancements have enabled the integration of complex electronic devices into single fibers, fostering the development of flexible and wearable fiber-based systems [61, 62]. High-resolution feature definition has also been achieved on textile platforms, supporting the mechanical flexibility and porosity of the fabrics [63, 64]. For instance, Hu et al. created an all-in-one fiber supercapacitor by selectively reducing graphene oxide on a fiber to form electrode lines using laser writing [65]. Nguyen et al. developed serially integrated supercapacitor units on a monofilament fiber using laser micromachining, allowing precise control over electrode placement and expanding the operating voltage range (Fig. 2d) [66]. Additionally, Ren et al. directly nano printed a 3D achromatic diffractive metalens onto the end facet of a single-mode fiber. This structure, fabricated using two-photon polymerization with a femtosecond laser, showed broadband polarization-independent focusing on the near-infrared telecom range [67].

Most patterning techniques for electronic devices were initially developed for flat 2D substrates. Thus, developing specialized equipment for curved or 1D geometries is crucial for achieving precise and reliable patterning. These advancements will play a significant role in enhancing high-resolution fabrication strategies for next-generation fiber-based electronic systems.

### Spinning Techniques for Fiber Shaping and Functionalization

Fiber spinning is a versatile technique for producing single-stranded fibers and complex structures. This method encompasses approaches such as electrospinning and wet spinning, enabling continuous fiber production with controlled compositions and geometries. The process involves extruding spinning fluids (polymer melts or solutions) through a spinneret nozzle, resulting in fibers that solidify by cooling or solvent evaporation. Effective spinning fluids must show fluidity and cohesion for stable fiber formation. Solution-based spinning methods include electrospinning, wet spinning, dry spinning, jet spinning, and microfluidic spinning [68, 69]. In addition, spinning techniques, particularly wet and melt spinning, are already proven in industrial fiber manufacturing, supporting high-throughput, continuous production of functional fibers with tunable structure and material composition, while electrospinning offers niche advantages in nanoscale morphology control for high-sensitivity applications.

Electrospinning employs an electric field to draw charged polymer solutions or melts from a spinneret, producing fibers in the nanometer range, as illustrated in Fig. 2e(1). This technique yields fibers with high surface area, porosity, and structural tunability, making them suitable for sensor applications due to their flexibility and nanoscale morphology [70, 71]. By adjusting solution concentration, viscosity, and rotation speed, film thicknesses ranging from tens of nanometers to tens of micrometers can be achieved [72]. However, electrospinning is limited by low production rates and dependency on conditions like applied voltage and flow rate, which affect fiber quality. For instance, Cao et al. developed a coaxial-fiber supercapacitor with enhanced folding resistance by electrospinning a polyacrylonitrile nanofiber mat onto a fiber electrode, resulting in a separator layer with tunable thickness (0.4–4 μm), high porosity and strong adhesion [70].

Wet spinning is a traditional method where a polymer solution is extruded through a spinneret into a coagulation bath, solidifying fibers via solvent exchange or evaporation (Fig. 2e(2)). This technique allows for thicker fibers than electrospinning and is compatible with various natural and synthetic materials [73, 74]. Its high productivity supports large-scale manufacturing, enhancing cost-effectiveness. However, wet spinning has limitations in minimum fiber size and material compatibility. Duan et al. developed stretchable and conductive fiber strain sensors by combining thermoplastic polyurethane with deep eutectic solvents to form a polymeric network within the fiber matrix [75]. These flexible fibers were produced using a simple wet-spinning and ultraviolet dual-curing process, showing the scalability and practicality of this method. Chen et al. introduced a wet-spinning strategy using ionic additives to enhance the conductivity and stretchability of PEDOT-PSS fibers, improving mechanical integrity and overcoming conventional processing limitations [76].

To provide a clearer quantitative comparison among fabrication techniques, Table 2 summarizes representative fabrication process metrics such as layer uniformity, yield, and mechanical endurance based on recent literature reports.

**Table 2:** Comparative summary of fabrication process metrics

Fabrication technique	Key features	Thickness	Reliability	Endurance	Refs
Coating	Low cost, scalable, compatible with reel-to-reel systems	1 m fiber length, 50 µm diameter	> 90% coating uniformly on fibers	–	[77]
		5 cm ± 5%-10% variation	> 90% coating on smooth fibers	Withstand > 103 bending cycles at 10% strains	[78]
Thermal drawing	Kilometer-scale production; precise multi-material integration	110 µm, 390 µm fiber diameter, 1 cm yarn	> 95% yield for multi-core yarn	Withstand 75 cycles at 200% strain	[79]
		350 µm fiber diameter, 200 cm fiber	> 85% yield for multi-core fibers	Stable up to > 105 cycles at 20% strain	[80]
Sputtering (PVD)	Dense metallic films, precise control over stoichiometry	100 µm diameter	~ 80% homogeneity	> 104 cycles at 20% strain	[81]
		10–500 nm; homogeneity	~ 85%–95% reproducibility	Film adhesion failure after > 103 cycles unless encapsulated	[82]
Atomic layer deposition (ALD)	Excellent conformality, dielectric control	7–10 µm thick	–	Stable up to 104 flexible cycles at 30% strain	[83]
		1–100 nm fiber length	> 98% reproducibility;	Maintains insulation up to 104 cycles	[84]
Spinning (Wet/Electrospinning)	Tunable porosity	90 mm Fiber diameters	> 85% yield for uniform yarn	Up to 103 at 50% strain	[85]

Although each fabrication technique offers distinct advantages, current research often treats these processes in isolation, resulting in a disconnect between material preparation, device fabrication, and system-level integration. Future manufacturing of SoF technologies show transition toward process convergence and hybrid integration, where multiple fabrication routes are harmonized into a unified production pipeline. This will involve the development of modular, reel-to-reel manufacturing platforms that sequentially combine deposition, patterning, and encapsulation on continuous fiber strands, as well as digital-twin based process control to ensure reproducibility and precision across each step. The incorporation of AI-driven feedback systems further enables real-time optimization of coating uniformity, device yield, and minimizing variability across large production volumes. Ultimately, the future direction lies in establishing an interoperable, data-informed manufacturing ecosystem that bridges the gap between laboratory-scale fabrication and industrial-scale production, transforming discrete processing steps into a continuous, adaptive, and intelligent manufacturing framework for next-generation SoF systems.

To provide clearer technical insight into the implementation of SoF technologies, the performance variations of representative materials under specific fabrication conditions are discussed. For conductive polymer coatings (e.g., PEDOT: PSS, PEDOT-CNT hybrids), coating thickness (200 nm–5 µm) and annealing temperature (80–180 °C) strongly affects conductivity, with mobility improvements observed when solvent evaporation is optimized and residual stresses are minimized. In thermally drawn multi-material fibers, changes in temperature (± 2–5 °C) significantly influence polymer phase alignment and residual stress, yielding variations of up to 20% in electrical resistance and > 30% in mechanical endurance across fiber lengths. For metal nanowire or CNT composite coatings, solvent polarity and dispersion concentration impose percolation network density, leading to strain-dependent resistance variations from Δ*R*/*R*_o_ ≈ 10%–40% after 1,000 bending cycles. In ALD-based dielectric layers, increasing cycle number or substrate temperature improves barrier density but becomes brittle; films grown at lower temperatures maintain flexibility but show ~ 15% higher leakage current under mechanical strain. Liquid metal channels (EGaIn, Galinstan) exhibit stable conductivity only when injection pressure, oxide layer thickness, and channel geometry are properly controlled, with Δ*R*/*R*_o_ kept below 5% after 1,000 cycles in optimized systems. This demonstrates the processability parameters directly modulate microstructure, interfacial adhesion, and mechanical robustness, eventually determining device-level performance and long-term SoF reliability.

## Integrated Electronic Components in Fiber Platforms

This section presents an overview of the functional components of fiber-based electronic systems, including transistors, memory, and artificial synapses. These components have been reengineered to meet the geometrical and mechanical constraints of fiber substrates, enabling intelligent, responsive, and reconfigurable functionalities in wearable electronic textiles. Electronic circuits consist of various components: passive elements (resistors, capacitors, inductors), active elements (diodes, transistors), connectors (headers and cables), power sources, sensors, and actuators. These serve as fundamental building blocks for a range of applications. Microcontroller-based platforms such as Arduino often function as central control units. Most existing electronic circuits are on 2D planar substrates, which limit their use in wearable technologies. By contrast, 1D fiber-based devices offer significant advantages for next-generation applications owing to their seamless integration with textiles. The high aspect ratio of the fiber structures enhances flexibility, allowing them to conform effectively to irregular skin surfaces and accommodate complex human motion. This adaptability makes fiber-based circuits promising for wearable systems that require stable performance under continuous deformation and dynamic stress [86].

### Sensing and Perception Units

Energy-efficient sensor-computing systems for edge applications have recently gained traction in real-time environmental and physiological data collection. Sensor modules serve as key components in fiber-based integrated, converting mechanical, thermal, optical, and biochemical signals into electrical data [87, 88]. They enable seamless signal acquisition, processing, and transmission, allowing smart fiber-based systems to adapt dynamically to their surroundings. Combining multiple sensing elements into a single platform enhances spatial resolution, sensitivity, and multifunctionality in smart textiles [89–91]. Integrating diverse sensor types such as temperature, pressure, motion, biochemical, and optical sensors into dense, addressable arrays captures a wide range of signals across different regions of a textile. Additionally, pairing sensors with ICs improves data accuracy, real-time monitoring, and feedback mechanisms. These advancements in stretchable architecture and low-noise signal processing have led to high-density sensory arrays that can seamlessly connect with fiber-based electronics [92–94]. Collectively, these innovations enable intelligent and responsive e-textile systems with expanded capabilities and user comfort.

#### Mechanical Sensors for Tactile and Motion Detection

Mechanical sensors are extensively used in tactile feedback applications, using electrical, pneumatic, deformative, and vibrational stimuli [95]. These sensors enhance the intuitiveness of interactions, enriching virtual and augmented reality experiences. Mechanical sensors can offer valuable insights into medical diagnostics by integrating data on body movements and environmental conditions. Current research focuses on wearable systems that LEDs with pressure sensors for visual feedback and exact tactile sensing [96, 97].

Haptic-sensing textiles are classified into capacitive and piezoresistive types [98]. Capacitive sensors detect touch through changes in capacitance while piezoresistive sensors alter their resistance under pressure. Incorporating conductive nanowires or nanoparticles into elastomeric matrices improves the stretchability and conductivity of sensing fibers [99]. Additionally, porous architecture enhances mechanical compliance, with common materials including carbon nanotubes, polymer composites (waterborne polyurethane and polyvinyl alcohol), PEDOT: PSS, and PVDF-TrFE [100–103]. Organic- and carbon-based materials often show limited thermoelectric performance, while inorganic materials, despite higher efficiencies, are less suitable for stretchable applications due to their rigidity.

**Fig. 3:**
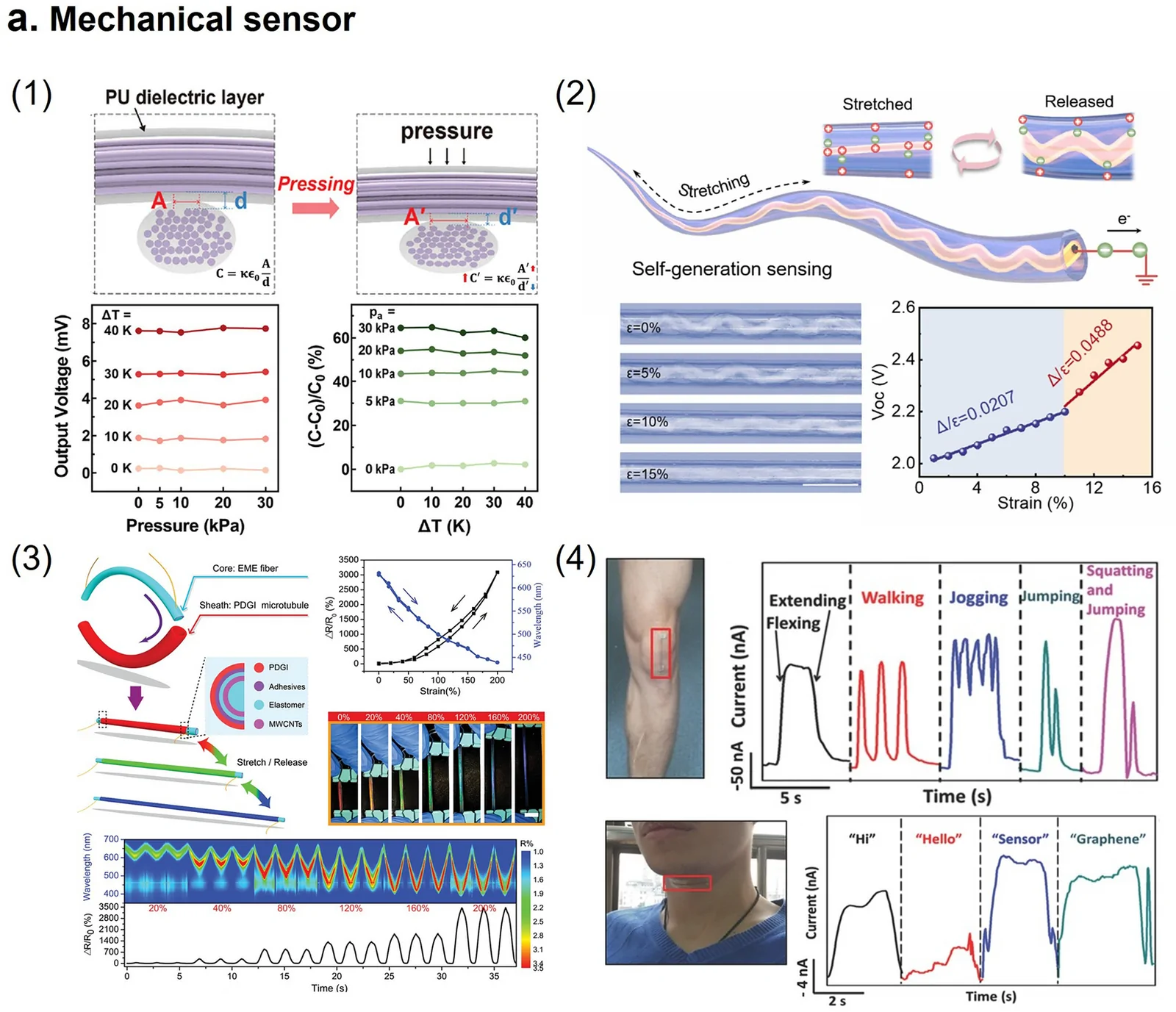
Sensing Units on/in fiber. **a** Mechanical sensor. (1) Working principle of the capacitive CuI fiber-based pressure sensor and changes in the output voltage (*V*_out_) and relative changes in capacitance ((*C*–*C*_o_)/*C*_o_)) of the CuI fiber-based pressure sensor under simultaneous stimuli of various pressures and temperature differences (Δ*T*). Reprinted with permission from Ref. [106]. Copyright 2024, Wiley–VCH GmbH. (2) Working mechanism and electrical performance of self-powered interactive fiber electronics with photographs of the fiber stretched from the original state to 15% strain. Scale bar: 500 µm; and linear relation between the open-circuit voltage (*V*_OC_) and the strain of the fiber Reprinted with permission from Ref. [107]. Copyright 2023, Elsevier. (3) Schematic diagram showing the fabrication of interactive full-color changeable multi-sheath interactive fiber strain sensor; Relative resistance and the reflection wavelength curve as a function of strain under cyclic stretching/releasing up to 200% strain; optical images of the sensor under different strains with Scale bar of 1 cm; relative resistance and reflection peak wavelength under different strains of 40%, 80%, 120%, 160%, and 200% at a frequency of 0.5 Hz. Reprinted with permission from Ref. [110]. Copyright 2020, Wiley–VCH GmbH. (4) Wearable sensor attached to the knee, marked in the red box; Responsive curves of wearable sensor on the knee under motions of flexing/extending, walking, jogging, jumping, and squatting-jumping; Wearable sensor attached to the throat, marked in the red box; Responsive curves when wearer spoke “Hi,” “Hello,” “Sensor,” and “Graphene.” Reprinted with permission from Ref. [114]. Copyright 2015, Wiley–VCH GmbH

Studies have developed stretchable fiber sensors from thermoelectric materials capable of detecting strain and temperature by sensing changes in contact area under pressure, modulating their capacitance or resistance, as illustrated in Fig. 3a(1) [104, 105]. Yoon et al. proved a multimodal sensor using thermoelectric fibers with dense CuI nanoparticles, achieving a maximum strain of approximately 83.5% and a Seebeck coefficient of 203.6 µV K^−1^ for simultaneous temperature, strain, and pressure detection [106]. Wang et al. introduced a triboelectric fiber capable of internal self-generation and external contact electrification, manufactured at speeds exceeding 20 m min^−1^ via spinning (Fig. 3a(2)). The helical structure of the fiber enables energy harvesting and strain sensing, showing two sensitivity regimes: a lower sensitivity up to 10% deformation (Δ/*ε* = 0.0207) and higher sensitivity beyond 10% (Δ/*ε* = 0.0488) [107].

Mechanical sensors connect with transducers to digitize signals for control, monitoring, and measurement applications. For instance, pressure sensors can indirectly detect humidity via material swelling [108, 109]. Wang et al. developed an interactive fiber sensor combining a piezoresistive fiber with photonic microtubules for mechano-chromic color change, providing electrical signal readout and full-color visual response (Fig. 3a(3)). With high sensitivity (gage factor ≈ 24.2), fast response, and high resolution (≈ 1 nm%), this sensor enables real-time tactile and visual feedback [110].

Piezoresistive and piezo capacitive sensors also enable voice and motion sensing by capturing subtle vibrations of the vocal cords [111, 112]. Cheng et al. developed a graphene-based fiber sensor using a double-covered yarn structure with a polyurethane core and polyethylene outer layer (Fig. 3a(4)). These fibers were responsive to stretching, bending, and twisting, with a low detection limit (0.2% strain), wide sensing range (up to 100%), rapid response time (< 100 ms), and long-term durability (stable over 10,000 cycles). The sensor effectively recognizes words such as “Hi,” and “Hello,” with high accuracy [113, 114].

#### Electrochemical Sensors for Biochemical Monitoring

Electrochemical sensors integrated into fibers create lightweight, comfortable wearable monitoring systems. These fiber-based sensors enable the continuous real-time tracking of physiological parameters, offering precise, personalized healthcare [115, 116]. By transducing biochemical stimuli into electrical signals, they can accurately detect key biomarkers such as pH, glucose, and lactate, easing the dynamic assessments at the molecular level [117]. For instance, Ji et al. developed a thread-based pH sensor within a smart bandage for chronic wound monitoring, featuring Bluetooth connectivity for real-time tracking via smartphones [118]. Advances in sensor technology, miniaturization, power efficiency, and wireless communication are expected to enhance the functionality of next-generation fiber-integrated systems, leading to intelligent electronic textiles [119, 120].

**Fig. 4:**
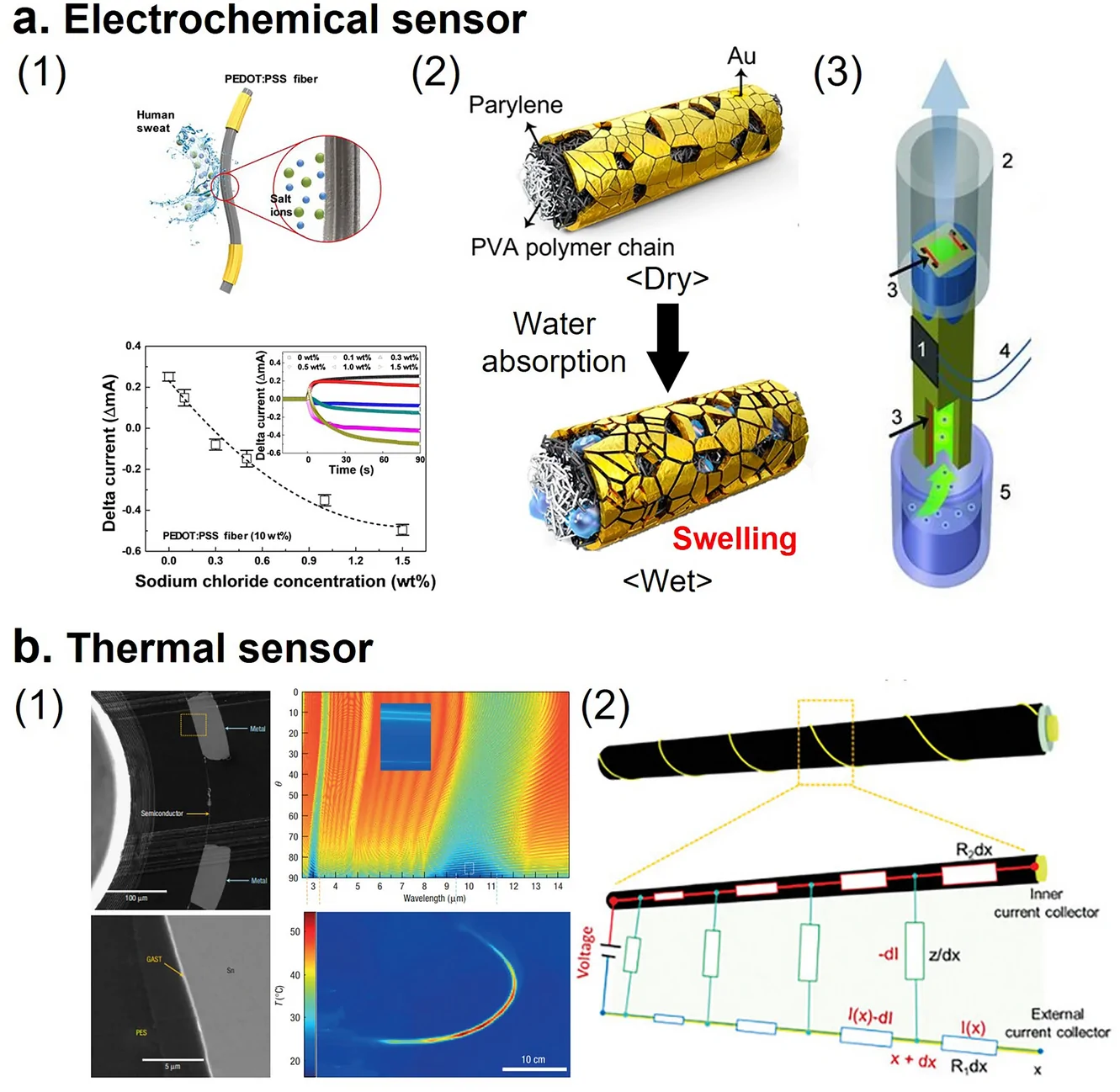
Sensing Units on/in fiber of **a** Electrochemical sensor. (1) Schematic for the formation of the PEDOT: PSS fiber and changes in current depending on the concentration of sodium chloride in water. Reprinted with permission from Ref. [123]. Copyright 2019, Springer Nature. (2) Schematic of the working principle showing the change in the polymer structure in response to water absorption. Reprinted with permission from Ref. [125]. Copyright 2019, American Chemical Society. (3) Schematic of chemiluminescent measurement setup. Reprinted with permission from Ref. [130]. Copyright 2012, Wiley–VCH GmbH. **b** Thermal sensor. (1) Thermal, electrical and optical properties of the hybrid fiber. Reprinted with permission from Ref. [143]. Copyright 2005, Springer Nature. (2) Equivalent circuit diagram per unit length of the fiber. Reprinted with permission from Ref. [145]. Copyright 2024, Wiley–VCH GmbH

Sweat Sensing: Wearable electronics must work in dynamic microclimates where temperature, humidity, and sweat composition vary. Sweat is a crucial biofluid for thermoregulation and metabolic waste excretion, including minerals and electrolytes. Excessive electrolyte loss can lead to dehydration and imbalance, making sweat a valuable target for non-invasive health monitoring [121, 122]. Sweat sensors employ ion-selective electrodes or capacitive sensing in which ionic interactions with the sensing layer generate quantifiable electrical signals. Lim et al. introduced a PEDOT: PSS fiber sweat sensor fabricated using conventional wet spinning (Fig. 4a(1)). The conductivity proved to be a linear response to sodium chloride concentration in the liquid environment [123]. Specifically, the I–V characteristics were checked after the fibers reached electrochemical equilibrium with a sodium chloride solution, enabling exact sensitivity quantification.

Humidity Sensing: Humidity sensors are essential in integrated wearable systems, particularly for physiological signal acquisition and environmental mapping. Semiconducting polymers are promising for humidity sensing owing to their responsiveness to water molecule adsorption, which modulates their electrical conductivity [124]. In fully integrated systems, these sensors enable multimodal health tracking, alongside temperature, strain, and biochemical parameters. Jeong et al. developed a nano mesh humidity sensor (Fig. 4a(2)) designed for real-time skin moisture monitoring. Made from breathable, biocompatible materials, like gold, PVA, and parylene C, the device offers high gas permeability and mechanical compliance for extended wear without skin irritation [125]. It has also been effectively used for long-term monitoring of relative humidity on plant surfaces, with potential applications in human physiological monitoring during physical activity or disease.

Gas Sensing: Detecting disease-related volatile biomarkers (e.g., acetone for diabetes, trimethylamine for trimethylaminuria, and ammonia for renal disease) poses challenges owing to low concentrations, intermittent release, and sensory adaptation [126, 127]. Fiber-based gas sensors provide solutions by offering conformal interfaces for real-time sampling, improved wearability, and multianalyte capability through functionalized fiber arrays. However, conventional sensors confined to one side of the fiber suffer from reduced sensitivity due to restricted surface area for gas interactions [128, 129]. To address this, Gumennik et al. introduced a monolithic design integrating photo detectors (PDs) along a hollow fiber core, allowing for active gas circulation (Fig. 4a(3)). This system achieved a sensitivity of 0.176 ± 0.005 nW ppm^−1^ and a noise equivalent power of 0.731 nW Hz^−0.5^, with a detection limit as low as 10 ppb for peroxide vapor [130]. Thermal treatment further enhanced its chemiluminescence efficiency.

For benchmarking standardizations, comparative summary of conventional planar device and fiber-based sensor device are discussed: for sweat sensing, fiber-based electrochemical sensors utilizing Ni, Co, or MXenes-modified fibers typically achieve sensitivities of 40–180 µA mM^−1^ cm^−2^ and detection limits of 0.1–5 µM for glucose, comparable to those of planar screen-printed electrodes (20–100 µA mM^−1^ cm^−2^; LOD ≈ 1–10 µM) [131, 132]. Unlike rigid substrates, fiber sensors maintain > 95% signal retention after 1000 bending cycles, highlighting their mechanical robustness and conformal contact with skin.

In gas sensing, fiber devices based on SnO_2_, WO_3_, or polyaniline nanocomposites exhibit response values (*R*_g_/*R*_a_) of 20–60 for 10 ppm NH_3_ or NO_2_ at room temperature, with response/recovery times of 5–20 s, outperforming many planar thin-film sensors that typically require elevated operating temperatures (150–300 °C) to achieve comparable sensitivity. Moreover, coaxial and porous fiber geometries enhance surface area-to-volume ratios, enabling faster diffusion and lower detection limits (to ppb level) while retaining signal stability under twisting or stretching [133, 134]. These results demonstrate that fiber sensors approach surpass planar device performance while offering unique mechanical adaptability, washability, and integration compatibility with textiles.

#### Temperature Sensors for Thermal Management

Temperature sensors are crucial in fiber-based electronic systems, especially for real-time monitoring in extreme environments. The integration into fibers is gaining traction for applications like vital-sign tracking, smart garments, and fire-detection [135–137]. These temperature-sensitive devices convert thermal fluctuations into electrical signals, enabling early warnings to prevent disasters like fires or overheating of critical systems [138–140]. Effective thermal management is crucial for ensuring the stability and longevity of computing platforms, as accumulated heat from processors can lead to degradation or failure if unmanaged. Radiative cooling has appeared as a promising solution. When temperature sensors are integrated with thermally insulating or radiative cooling structures, they can autonomously detect thermal anomalies and trigger proper countermeasures. This closed-loop system enhances reliability through intelligent temperature regulation, activating cooling when thresholds are exceeded [141, 142].

Bayindir et al. developed a multifunctional fiber with optical, electrical, and thermal capabilities that allows for self-monitoring optical transmission along its length (Fig. 4b(1)). Thermal sensors near the hollow core detect localized temperature changes, generating electrical signals based on the exponential temperature sensitivity of the semiconductor [143]. This setup provides real-time diagnostics for abnormal heating or material defects during optical communication.

Furthermore, advanced designs now include active thermal regulation. Wang et al. proposed a radiative electrochromic fiber with a helically wound external electrode and infrared responsive coating (Fig. 4b(2)). This textile proved enhanced thermal stability by dynamically modulating infrared radiation in response to temperature fluctuations [144, 145]. When applied to simulated skin models, the fiber-maintained temperature variation within approximately 1.6 °C under ambient temperature swings of 11.2 °C, outperforming traditional textile materials, which showed larger fluctuations of about 2.9 °C. This proves the potential of electrochromic radiative fibers for achieving energy-efficient, thermally adaptive wearables [146, 147].

#### Bioelectrical Sensors for Physiological Signal Monitoring

Continuous real-time monitoring of cardiovascular health is essential in modern healthcare. Among bio-signal modalities, electrocardiogram (ECG) sensing is critical for diagnosing arrhythmias, myocardial ischemia, and other cardiac disorders [148]. However, conventional rigid electrodes and adhesive-based sensors suffer from poor skin conformity, motion artifacts, and limited reusability, hindering their long-term use in wearable applications. To address these limitations, fiber-based electronics offer mechanical flexibility, compatibility with textile platforms, and unobtrusive high-fidelity bio-signal acquisition [149, 150]. Fiber-shaped sensors embedded in electronic textiles (e-textiles) provide a seamless, washable, and scalable route for continuous ECG monitoring, ensuring comfort without sacrificing performance.

**Fig. 5:**
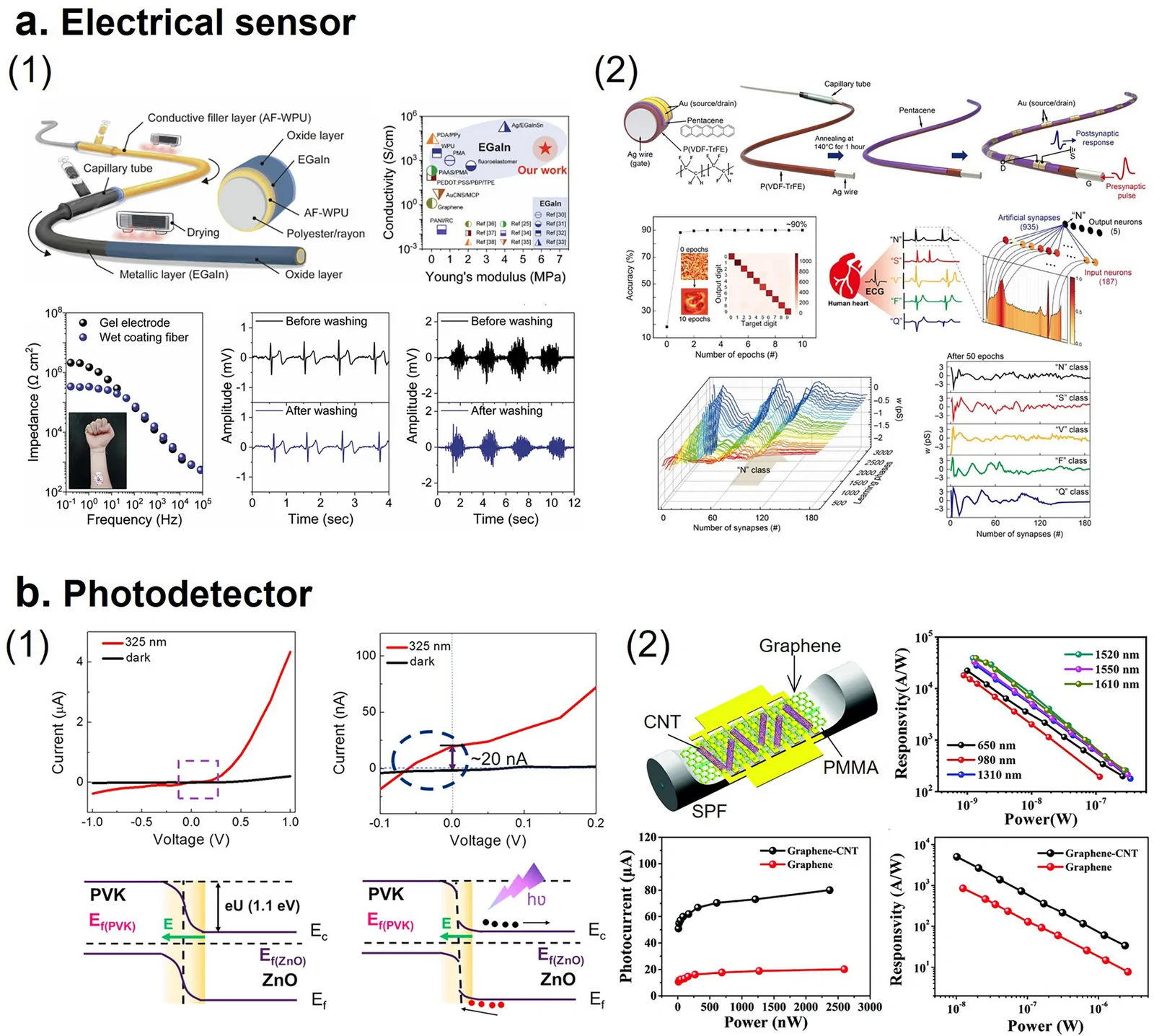
Sensing Units on/in fiber. **a** Electrical sensor. (1) Manufacturing process of CTFs, comparison between conductivity vs Young’s modulus of CTFs from previous works, comparison of impedance between the gel electrodes and wet CTFs, thereby showing the impedance values (< 10^6^ Ω cm^2^); ECG and EMG measurements using the CTFs before and after washing. Reprinted with permission from Ref. [151]. Copyright 2025, Springer Nature. (2) Fabrication process and schematics of the 1D organic artificial multi-synapses; Recognition simulation for MNIST and ECG patterns of fiber-shaped ferroelectric organic transistors. Reprinted with permission from Ref. [152]. Copyright 2020, AAAS. **b** PD. (1) Current–voltage curves in dark and under 325 nm laser (0.38 mW cm^−2^); Energy band diagrams of the *p–n* junction between PVK and ZnO at 0 V in the dark and under UV light. Reprinted with permission from Ref. [157]. Copyright 2016, Elsevier. (2) Carbon nanotube–graphene all-fiber; photocurrent and photoresponse of the AFIP with and without carbon nanotubes photocurrent and responsivity of the AFIP as a function of light power with (black lines) and without (red lines) CNTs at 1550 nm. Reprinted with permission from Ref. [158]. Copyright 2020, Royal Society of Chemistry

Kim et al. developed multilayered conductive tough fibers (CTFs) using a continuous CTAC process (Fig. 5a(1)). This fiber consists of a polyester-rayon core for strength, a silver flake–waterborne polyurethane (AF–WPU) composite layer for conductivity and adhesion, and an outer eutectic gallium–indium (EGaIn) coating that ensures ultrahigh conductivity (~ 6.42 kS/cm) and water resistance via native oxide stabilization. This multilayer configuration imparts high stretchability (up to 70%), moderate mechanical stiffness (Young’s modulus ≈ 6.22 MPa), and excellent environmental durability, supporting performance after over 100 wash cycles and 24 days of submersion (IPX8 rating). The CTFs exhibited skin–electrode impedance levels at ~ 20 Hz, comparable to commercial gel electrodes, and achieved a signal-to-noise ratio of 40.3 dB during ECG recording [151]. These robust characteristics were supported even after repeated bending, laundering, and underwater operations, underscoring the potential of this material for sustainable long-term e-textile ECG monitoring.

Ham et al. introduced a neuromorphic textile platform composed of 1D organic multi-synaptic fibers that integrate bio-signal acquisition with in-fiber signal processing (Fig. 5a(2)). Built on 100-µm-diameter silver wires, the platform incorporates ferroelectric P(VDF-TrFE) gate dielectrics and pentacene semiconductors to realize fiber-shaped ferroelectric organic transistors. These devices emulate synaptic behaviors such as short-term and long-term potentiation and depression through electrically tunable conductance states [152]. Crossbar arrays are constructed using orthogonal Ag wires function as NOR-type synaptic networks, supporting stable performance across 6,000 switching cycles under strain (bending radius of 2.5 mm). When deployed in a single-layer neural network, the synaptic fiber arrays achieved approximately 70% classification accuracy for five arrhythmic ECG waveforms. This highlights the potential of neuromorphic e-textiles not only for bio-signal monitoring but also for real-time on-device learning and classification without reliance on external processors.

#### PDs for Optical Sensing and Communication

PDs are essential components of ICs that convert optical input into electrical outputs, enabling a wide range of advanced functions in modern electronic systems. They are particularly ubiquitous in telecommunications, where they often take the form of semiconductor *p*–*n* junction diodes in contact with adjacent electrodes. A simpler alternative is a photoconductor, whose conductivity varies in response to the incident light intensity [153, 154].

PDs are crucial in ICs for applications such as high-speed data communication, optical interconnects, and optical sensing systems. Their compact semiconductor-based structure eases seamless integration into the circuit architecture, improving spatial efficiency while improving signal processing speed and energy consumption. Additionally, their compatibility with CMOS manufacturing processes allows for scalable and cost-effective production, making them well suited for real-time light detection in next-generation optoelectronic platforms. Precise control of carrier generation and recombination in *p*–*n* junctions or photoconductive layers ensures robust and reliable operation across various environments, including consumer electronics, industrial systems, and biomedical devices [155, 156].

Unlike their planar counterparts, fiber-shaped PDs often struggle to achieve self-powered functionality because of their geometric curvatures and limited surface areas. These constraints typically require the use of external power sources for autonomous sensing. However, this limitation was effectively addressed by Dong et al., who developed a wearable omnidirectional ultraviolet PD capable of simultaneously serving as both a power source and a sensing unit (Fig. 5b(1)). Their design employed a Zn wire substrate processed via hydrothermal growth and dip-coating to form crystalline ZnO nanowires layered with PEDOT: PSS and PVK. The resulting *p-n* heterojunction structure enabled zero-bias operation with an *I*_light_/*I*_dark_ ratio of approximately 2 and a responsivity of 9.96 mA W^−1^ at 350 nm [157]. The device also kept stable performance under mechanical deformation and enabled 360° light detection, showing a significant advancement in integrating energy harvesting with photodetection in fiber-based formats. Zhuo et al. introduced an all-fiber PD fabricated by wet transferring a graphene/PMMA film onto a structured polymer fiber (SPF) (Fig. 5b(2)). Intense light–matter interactions between the CNT/graphene hybrid films enabled high photoresponsivity across a broad spectral range (650–1610 nm). At a wavelength of 1550 nm, the device achieved photoresponsivity up to approximately 1.48 × 10^5^ A W^−1^ and could detect light intensities as low as 91.5 pW [158]. This fiber-integrated detector provides a cost-effective and robust solution for distributed optical sensing and real-time power monitoring in optical communication systems, thereby improving both performance and reliability.

### Signal Processing and Logic Units

In fiber-based integrated systems, components are selected and configured to meet specific system goals and application domains. These systems include multiple functional electronic elements that ease complex operations. Core building blocks include transistors, diodes, resistors, capacitors, inductors, microcontrollers, and interface modules. ICs are central to these systems, merging components into compact platforms for advanced signal processing and control. Various circuit configurations, such as resistor–capacitor arrays and transistor-diode architectures (1 T 1D), including thin-film transistors paired with photodiodes have been developed. Basic logic units like inverters, ring oscillators, and signal transducers have also been proven. Recently, emerging architectures such as memory processing and memory accelerators have gained attention for enabling parallel computation and localized data management on fiber substrates. The integration of these advanced units enhances the computational capabilities of fiber-based systems, supporting next-generation applications in wearable electronics and distributed sensor networks [159, 160].

#### Transistors for Signal Switching and Amplification

Transistors are essential components in logic circuits, serving as the core elements for signal amplification and switching. They are fundamental to circuit design, data processing, and signal transmission across various electronic applications [161]. In fiber-based electronic systems, two main types of transistors are employed: field-effect transistors (FETs) and organic electrochemical transistors (OECTs). These transistors enable the integration of electronic functionality into flexible textiles, expanding their applicability in wearable devices that require efficient signal management [162]. FETs and OECTs integrated into fiber architecture enable the development of complementary circuits using *n*- and *p*-type transistors. This offers advantages such as low static power consumption, rail-to-rail voltage output, high noise margin, and high gain [163].

**Fig. 6:**
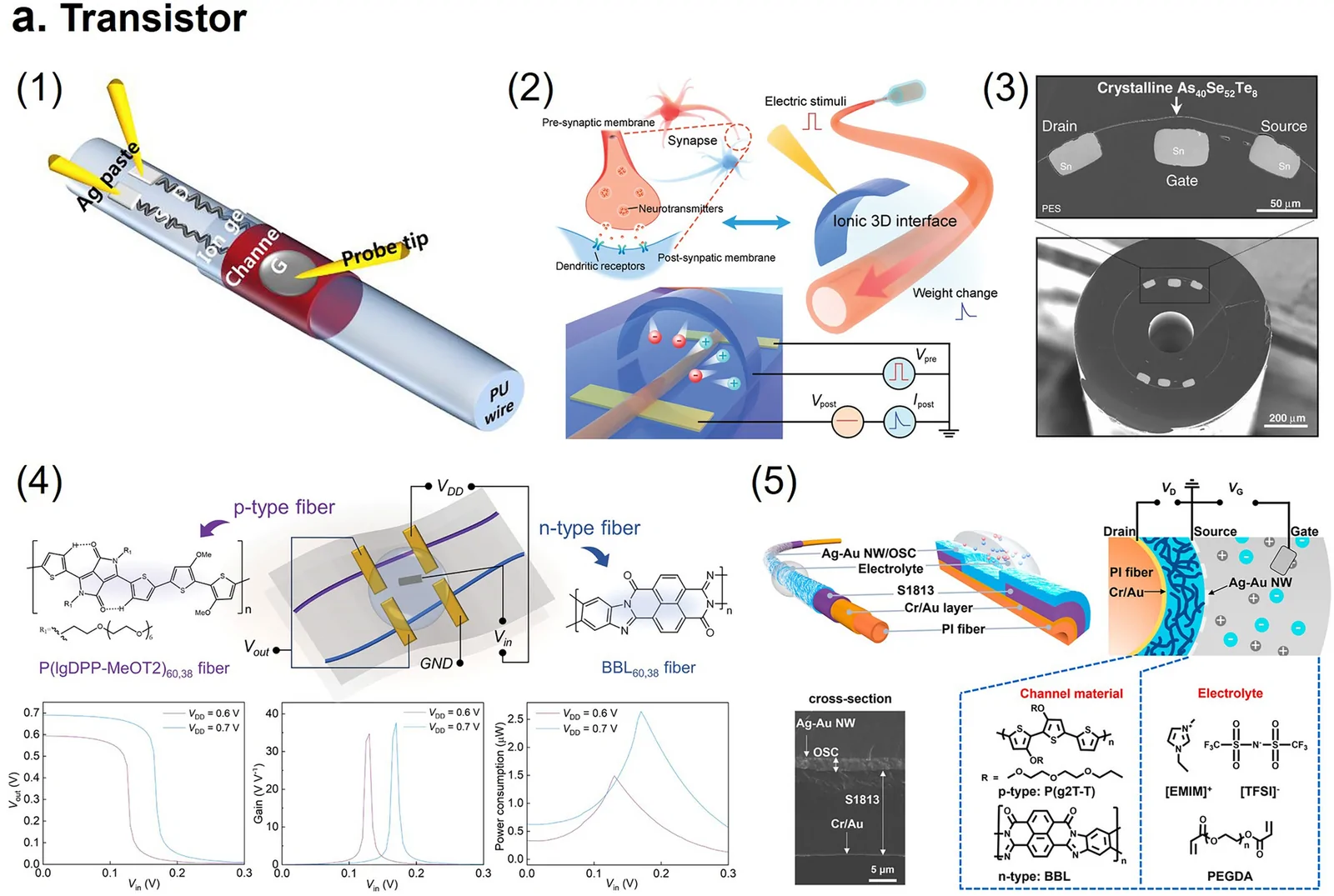
Processing Units on/in fiber. **a** Transistor (1) ~ (3): FET, (4), (5): OECT) (1) Schematic of fiber transistor based on buckled hybrid fiber electrodes. Reprinted with permission from Ref. [165]. Copyright 2019, Elsevier. (2) Schematic image of a biological synapse and PQT-12 synaptic transistor with 3D ionic electrolyte. Reprinted with permission from Ref. [166]. Copyright 2020, Wiley–VCH GmbH. (3) SEM image of the cross section of the fiber device whole structure (lower panel). Magnification of one of the two devices (upper panel). Reprinted with permission from Ref. [167]. Copyright 2010, Wiley–VCH GmbH. (4) Fiber-based complementary inverters and their performance; Schematic of the complementary-type inverter and chemical structures of *p*-type P(lgDPP-MeOT2) and *n*-type BBL polymers; Voltage transfer characteristics; voltage gains, and power consumptions of the fiber-based inverter at different supply voltages. The supply voltage (*V*_DD_) is 0.6 or 0.7 V. Reprinted with permission from Ref. [173]. Copyright 2024, Wiley–VCH GmbH. (5) Fiber-shaped vertical OECT architecture cross-sectional structure diagram; schematic illustrating the electrical connections and the pathways for ion migration in the fiber-shaped vOECT; SEM image and Molecular structures of *p*-type and *n*-type semiconductors and quasi-solid-state electrolyte components. Reprinted with permission from Ref. [174]. Copyright 2023, American Chemical Society

Traditional fiber-based FETs typically consist of interlaced warp and weft fibers, where warp fibers function as source and drain electrodes, and the weft fiber serving as the gate (Fig. 1, 2010). Most designs adopt a bottom-gate top-contact configuration, with semiconductor channels and gate insulators deposited on the gate fiber. When a gate voltage is applied, field-effect doping occurs, enabling digital logic processing. However, woven fiber-based architectures experience delamination or fracture at contact points under mechanical strain [164]. To mitigate these issues, innovative approaches include integrating all device layers onto a single monofilament fiber. For instance, Lee et al*.* showed hybrid transistors using a pre-strain and buckle strategy on elastic thermoplastic polyurethane fibers (Fig. 6a(1)) [165]. These transistors-maintained performance under 50% strain and over 1,000 cycles, achieving a charge carrier mobility of 1.74 cm^2^ V^−1^ s^−1^ and an on/off ratio of 10^4^.

To further improve mechanical robustness, Liu et al. develop an organic FET via single-fiber electrospinning featuring a solid ion gate architecture with a large interfacial area between the electrolyte and the fiber-shaped channel (Fig. 6a(2)) [166]. This design enables ultralow-power operation and synaptic behavior emulation, achieving approximately 3.9 fJ per spike with sub mV operating voltages. Key electrical parameters mobility, subthreshold swing, threshold voltage, and on/off ratio are crucial for optimizing FET performance. Danto et al*.* introduced a fiber-compatible FET fabricated via thermal drawing, using a phase-change glassy semiconductor alongside metal electrodes (Fig. 6a(3)), enabling in situ modulation of electrical properties for logic processing [167].

In contrast to FETs, which rely on charge modulation at the semiconductor and dielectric interfaces, OECTs function through volumetric ionic doping within a mixed-ionic electronic polymer channel [168, 169]. The ions from the electrolyte reversibly enter and exit the channel, modulating their conductivity in the bulk. This mechanism allows for high transconductance at low operating voltages, making OECTs ideal for bio-signal amplification and bio-interface applications [170, 171]. Polymer semiconductors in OECTs are mechanically compliant and biocompatible, supporting fabrications on deformable substrates. However, challenges stay in controlling channel morphology, improved crystallinity and charge transport alignment, and ensuring long-term operational stability [172]. Wang et al. addressed these challenges by employing wet spinning to produce highly crystalline and aligned fibers from the *n*-type polymer poly(benzimidazo benzophenanthroline) (BBL), yielding improved charge mobility and stability in water (Fig. 6a(4)). These fibers proven over 90% performance retention after 4,000 switching cycles. Complementary inverters combining BBL and *p*-type P(lgDPP MeOT2) fibers achieved a voltage gain of 38 V/V at 0.7 V with low power consumption [173]. To address layout limitations, Zhong et al*.* developed a vertical fiber OECT with photopatterning short channels along the fiber axis (Fig. 6a(5)). This coaxial structure, featuring sequentially deposited drain, semiconductor, and porous Ag Au nanowire source contacts, enabled channel lengths under 3 µm. The vertical OECT exhibited transconductance values of 41.10 mS (*p*-type) and 2.25 mS (*n*-type), on/off ratios exceeding 10^4^, and reliable switching under strain [174]. The complementary inverter based on vertical OECTs achieved a voltage gain of 66.5 V/V at 0.6 V, outperforming planar designs due to improved gate-channel coupling and reduced channel dimensions. These findings highlight the potential of transistor technologies for logic, amplification, and neuromorphic computing in fiber-based electronics.

#### Memory Devices for Data Storage and Retrieval

Memory devices are essential in IC systems, as they store and retrieve data, influencing the overall system performance and functionality. Smart fibers with embedded memory capabilities can autonomously process inputs and generate outputs, serving as vital links between sensing and feedback, fostering advancements in computing, healthcare, and soft robotics. Breakthroughs in materials science have shown various materials exhibiting memristive behavior, characterized by electrically tunable resistance in response to voltage or current stimuli [175, 176].

**Fig. 7:**
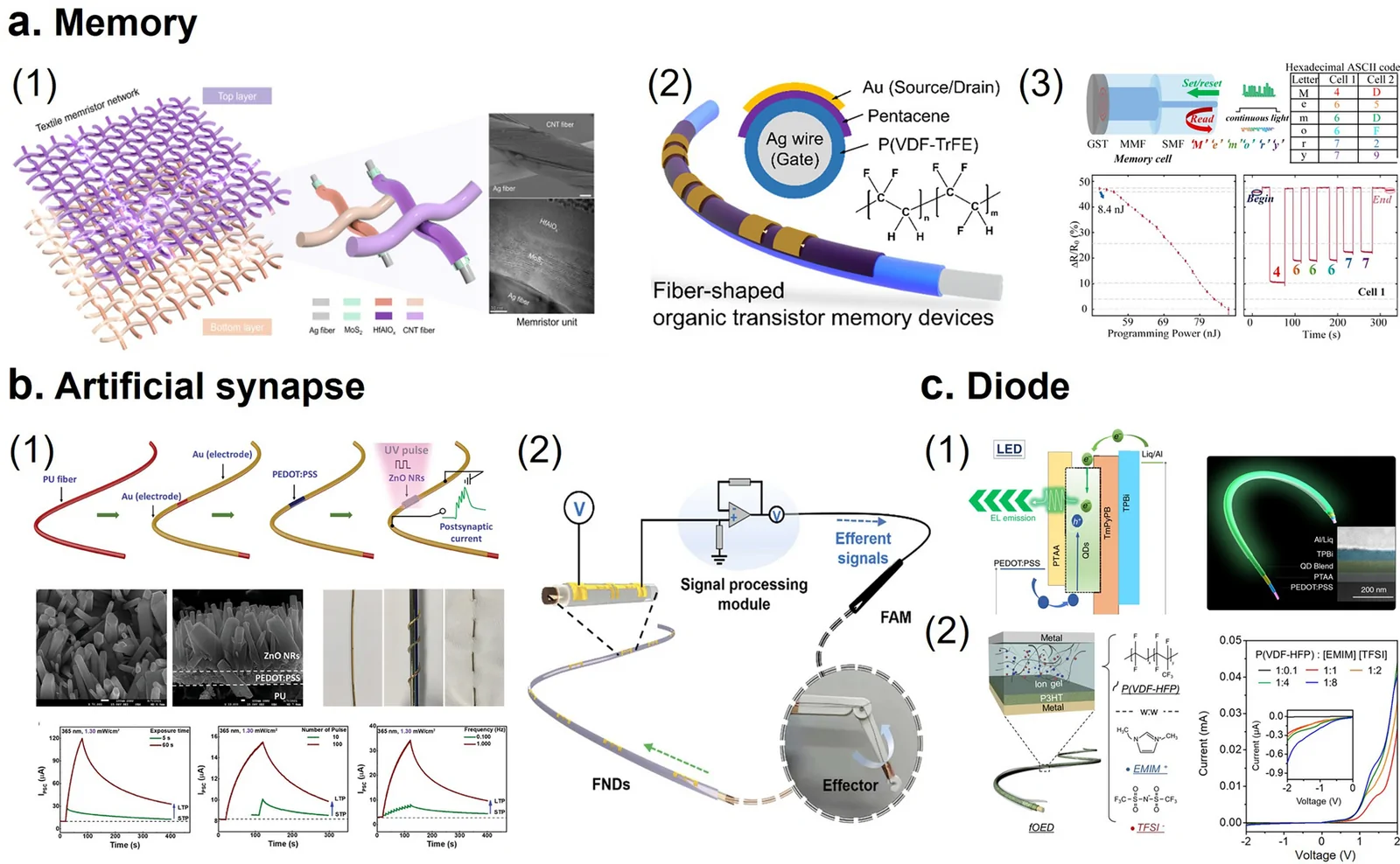
Processing Units on/in fiber. **a** Memory (1) Schematic image of textile memristor network, including top-layer device with synaptic plasticity and bottom-layer device with neural functions; structure of fiber-based memristor consisting of Ag/MoS_2_/HfAlO_x_/CNT; Scanning electron microscopy (top) and cross-sectional transmission electron microscopy (bottom) images of fiber-based memristor. Scale bar of top image, 40 μm. Reprinted with permission from Ref. [178]. Copyright 2022, Springer Nature. (2) Schematic of the device architecture of the Fiber Organic Memory. Reprinted with permission from Ref. [31]. Copyright 2019, American Chemical Society. (3) Application of fiber-integrated memory cells. Reprinted with permission from Ref. [186]. Copyright 2023, American Chemical Society. **b** Artificial synapses (1) Fabrication process of the FPAS; Top-view and cross-sectional view FE-SEM images of ZnO NRs/PEDOT: PSS heterojunction on a PU fiber; Optical images of as-fabricated FPAS, FPAS coiled on tubes, and FPAS sewn on fabric. Emulation of STP and LTP by spike-duration time-varied optical stimulus of the as-fabricated FPAS; the number of pulse-varied optical stimulus of the FPAS coiled on tubes; frequency-varied optical stimulus of the FPAS sewn on fabric. Reprinted with permission from Ref. [197]. Copyright 2024, Wiley–VCH GmbH. (2) Schematic illustration of the fibrous artificial neuromuscular system: FNDs, signal processing module, FAM, and effector. Reprinted with permission from Ref. [198]. Copyright 2022, Elsevier. **c** Diode (1) Schematic illustration of the working mechanism (LED mode) of the light-emitting/detecting bifunctional perovskite fiber and inset cross-sectional SEM image of perovskite fibers. Reprinted with permission from Ref. [205]. Copyright 2020, Springer Nature. (2) Schematic illustration of the fOED and I–V curves of the fabricated fOEDs at different ionic concentrations. Reprinted with permission from Ref. [207]. Copyright 2023, American Chemical Society

Memristors are appearing as promising two-terminal circuit elements and strong candidates for next-generation nonvolatile memory devices [177]. Their basic structure forming a top electrode (HfAlO_x_), active layer (Ag fiber, CNT fiber), and bottom electrode (MoS_2_) can be formed by weaving two conductive fibers (Fig. 7a(1)), enhancing their application in memory configurations [178]. These devices store program codes and operational data essential for CPU processing, improving computational speed and efficiency. Nonvolatile memories, such as flash memory, keep data without power for system recovery, while volatile memories, such as RAM, temporarily store data for immediate access. Logic operations use low-resistance states for logic ‘1’ and high-resistance states for logic ‘0,’ underscoring the foundational role of memory in logic circuits. Logic gates such as NAND can implement complete CPU architectures, highlighting the computational potential of memory [179]. Despite this promise, research on fiber-type memory devices, especially in wearable systems is limited. To date, only capacitive-type memory devices and fabric-based memories have been reported [180, 181]. Fiber- or textile-type memory configurations ease simple one diode–one resistor (1D–1R) and one selector–one resistor (1S–1R) architectures [104, 105], enhancing scalability and enabling in-memory computing [182, 183].

Various production methods such as electrochemical deposition (ECD), coating, and PVD have been developed for fabricating fiber-based memory [184, 185]. ECD alters the electrical properties of fibers by depositing ions or molecules onto their surfaces. For instance**,** Kang et al. showed fiber-shaped memory devices using organic transistor memories integrated into a metal wire (Fig. 7a(2)). These devices, which were fabricated on Ag microwires using a CTAC method, produced smooth and compact nanograined P(VDF-TrFE) films [31]. The resulting memory shown outstanding performance, characterized by high flexibility, reliable endurance over 100 cycles, and long-term retention (~ 5 × 10^4^ s) at low operating voltages (< 5 V). To achieve uniform thin films with controlled thickness and composition on fiber substrates, Liu et al. developed a fiber-integrated all-optical signal processing device via sputtering (Fig. 7a(3)). This device performs both storage and matrix–vector multiplication by connecting two memory units in parallel [186]. Using a specially designed Bessel-like light field, they achieved 19 discrete memory levels with low switching energy (90 nJ), sharp contrast ratio (47%), and fast single-pulse switching (200 ns). These features underscore the potential of all-optical processing units for data-centric applications, such as photonic neural networks and neuromorphic computing.

Compared with transistors and OECTs, research on fiber-integrated memory devices remains pivotal for fiber data storage and neuromorphic computation. Recent literature surveys include resistive RAM (ReRAM), ferroelectric, and ion-gel based memristors exhibit promising performance, but long-term reliability and scalability are still major bottlenecks. Typical fiber ReRAMs employ oxide layers such as HfO_2_, TiO_2_, or ZnO exhibit on/off ratios of 10^3^–10^5^, switching voltages of 0.5–2.0 V, and endurance up to 10^4^–10^5^ cycles, with performance degradability under repeated bending or humidity exposure. Ferroelectric fibers based on P(VDF-TrFE) or HfZrO_2_ films show polarization retention exceeding 10^4^ s and stable operation under 5%–10% tensile strain, showing their potential for flexible nonvolatile storage [178, 187, 188]. Similarly, iontronic or electrolyte-gated memristors leverage redox-active gels and polymer electrolytes to achieve analog tunability and fJ energy, enabling their use in low-power neuromorphic learning [189].

However, integrating such memory elements along a 1D fiber presents challenges in device-to-device variability, and scaling beyond 10^2^ units per meter. Hybrid designs that combine ferroelectric and ionic memory mechanisms show improved endurance (> 10^6^ cycles) and multi-level storage capability, offering high-density data fibers. Future directions on AI-guided materials screening defect switching kinetics, and coaxial fiber geometries that electrically isolate and stabilize memory arrays. Collectively, these advances will enable the development of mechanically robust, long-retention, and scalable memory fibers, sensing triads, processing, and storage needed for fully autonomous SoF platforms.

#### Artificial Synapses for Neuromorphic Computing

The concept of a bioinspired artificial retina or synapse on a fiber involves integrating biological principles into electronic systems for wearable and flexible applications. By mimicking biological synapses and auditory systems, these devices efficiently process complex data from the human body or environment. In e-textiles, crossbar-type architectures using 1D fiber devices enable artificial neural networks (ANNs) that support parallel signal processing and pattern recognition [152, 190].

Emulation of synaptic plasticity in neuromorphic systems relies on two approaches: analog and digital artificial synapses. Analog synapses, using memristive devices or ion-gated transistors, replicate continuous biological signal modulations through resistance or conductance changes. They excel in mimicking short- and long-term plasticity (STP/LTP) with low energy consumption, resembling learning mechanisms in biological networks [191–193]. In contrast, digital synapses use discrete states and spike-timing-dependent plasticity, employing CMOS-compatible circuits or multibit memristors for precise programmable updates. Analog systems focus on energy efficiency and biological fidelity, while digital implementations offer robustness against noise, easing seamless integration with conventional computing architectures [194, 195].

Implementing artificial synaptic functions on fibers is critical for wearable neuromorphic computing, enabling synaptic plasticity and learning. This development leads to intelligent wearable devices that can adapt to and interact with their environment. For example, bio-inspired artificial retinas on textiles can use photonic principles to process visual information, while artificial synapses ease adaptive learning and memory [196]. These systems can run autonomously using organic–inorganic heterojunctions and photogating effects, enhancing sensory data processing and real-time interactions.

Trung et al. developed a bioinspired artificial retina featuring a fibrous photonic artificial synapse composed of organic–inorganic heterojunctions (Fig. 7b(1)). This device runs in self-powered mode by capturing and releasing photogenerated carriers via photogating effects, allowing for autonomous function without external power sources. Additionally, fiber-based devices can replicate higher-order biological reflexes [197]. Ni et al. reported 1D neuromorphic fiber devices that mimic automatic reflexes and cognitive activities by emulating brain-like synaptic behaviors (Fig. 7b(2)). The devices supported synaptic weights under repeated electrical stimulation and mechanical bending, with pattern-recognition accuracy staying within 0.1% error during testing [198]. This underscores the potential of fiber-based artificial synapses for advancing biohybrid soft electronics and deep-learning neural network applications.

Textile neuromorphic devices are rapidly shifting toward device-level demonstrations to system prototypes, mostly persisting as proof-of-concept compared to conventional planar inorganic neuromorphic devices. Fiber or yarn-based synapses implemented with ferroelectric transistors, OECTs, iontronic gels, or memresistive junctions prioritize mechanical compliance, low-voltage operation, and direct textile integration over peak switching speed or endurance, enabling applications such as wearable sensor fusion, bio-interfaced learning, and soft-robotic reflexes. Technological advances include 1D ferroelectric multi-synapses woven into e-textiles for pattern learning, iontronic synapse fibers that drive textile sensorimotor loops at ultralow power, and fiber-memristor networks used for on-fiber reservoir computing collectively showing robust operation under bending and weaving with energy that is comparable to flexible planar organics yet generally below top-end Si/oxide memristors in speed/endurance [172, 178, 199]. Continued progress will hinge on materials/interface stability under humidity and washing, device-to-device variability control, and closed-loop calibration to keep synaptic weights during long-term wear. Typical fiber-type OECT or memresistive devices exhibit switching energies in the range of tens to hundreds of femtojoules per spike and on/off ratios of 10^2^–10^4^, values that are comparable to flexible planar organic devices but still fall short of the sub-femtojoule switching energies and > 10^6^ endurance cycles reported for Si-based or oxide memristors [200].

#### Diodes for Rectification and Logic Protection

Diodes are essential components in ICs, serving roles in rectification, voltage regulation, and switching. They ensure unidirectional current flow and protect against voltage spikes, enhancing system reliability [201].

Various diode types are employed depending on their electrical characteristics and target applications. The choice of diode type depends on circuit-specific parameters such as voltage rating, current-handling capacity, and switching behavior. Rectifier diodes ease AC-to-DC conversion in power supplies, while Zener diodes function as voltage regulators by supporting a fixed voltage. Light-emitting diodes (LEDs) convert electrical energy into light for displays and illumination and Schottky diodes are preferred in high-frequency power-conversion circuits due to their fast-switching speeds and low forward voltage drops [202, 203]. In IC designs, diodes are integrated for logical and protective functions, constructing logic gates, safeguarding sensitive nodes from electrostatic discharges (ESD), and enabling signal rectification. Advances in semiconductor manufacturing have miniaturized diodes to micron and submicron scales, enhancing circuit density and functionality [204].

Shan et al. developed a bifunctional fiber-based diode capable of light emission and detection, using perovskite quantum dots in a hybrid architecture. This simple, reproducible, solution-processed fiber (Fig. 7c(1)), proved narrow emission (FWHM ~ 19 nm) and enabled full-duplex light-fidelity (LiFi) communication, positioning it as a candidate for next-generation wearable systems [205]. Additionally, electrochemical and ionic diodes, which employ ion migration and redox reactions, enhance biocompatibility, and adaptability for skin [206]. Choi et al. introduced a fibriform organic electrochemical diode (fOED) with excellent rectifying properties (rectification ratio > 10^2^) and logical compatibility (Fig. 7c(2)). The fOED performed robustly under practical textile conditions, proving strong adhesion and durability during washing [207]. Furthermore, fOED-based circuits have been successfully implemented in textile-embedded logic gates and rectifiers, enabling AC-to-DC conversion and protection from transient electrical surges, improving the safety and reliability of wearable electronic systems.

### Interconnection Strategies in Fiber Electronics

Interconnects serve as physical conduits for transmitting digital signals and distributing power in ICs. As device miniaturization and integration densities advance, conventional interconnects are becoming significant bottlenecks in VLSI systems, particularly impacting input/output (I/O) performance and scalability [208, 209]. This challenge is further worsened in emerging platforms, such as fiber- and textile-based ICs, which require interconnects to have high electrical conductivity and mechanical flexibility to endure deformation during use [210].

Classical electrode materials, such as Au, Ag, and Cu, are typically more rigid than textiles or human bodies. Consequently, wearable electrodes must be engineered to endure human motion, moisture, and mechanical stress of deforming fabrics. Designing and interfacing electrodes with soft materials presents a major barrier to implementing electronic fibers [211].

Recent developments have focused on Liquid Metals (LM) like eutectic gallium-indium and metal nanomaterial networks to meet the demands of next-generation applications [212, 213]. Advanced fabrication techniques, including nanoscale coating, inkjet printing, and the integration of metal-polymer composites, are used to produce interconnects that combine robust electrical performance with the necessary flexibility for wearable and textile-based systems [214]. The rapid evolution of these materials and methodologies is central to overcoming traditional interconnect limitations and advancing rigid and flexible IC technologies.

There are four primary fabrication methods for conductive tracks in textiles: weaving, knitting, embroidering of conductive yarns, and patterning of conductive inks [208, 215]. Recent advancements have enabled the simultaneous formation of electrodes and other functional components, streamlined integration and enhanced the scalability of fiber-based electronic devices. For example, Liao et al. developed a solution extrusion method that allows for the simultaneous fabrication of electrodes and electrolytes, enabling continuous production of fiber batteries at an industrial scale [216]. This one-step process using an industrial spinneret simplifies integration and improves manufacturing efficiency. Seoane et al. introduced a stretchable and conductive carbon nanotube (CNT)-based paste for screen printing on a textiles substrate to create interconnectors between electronic instrumentation and sensitized garments [217].

**Fig. 8:**
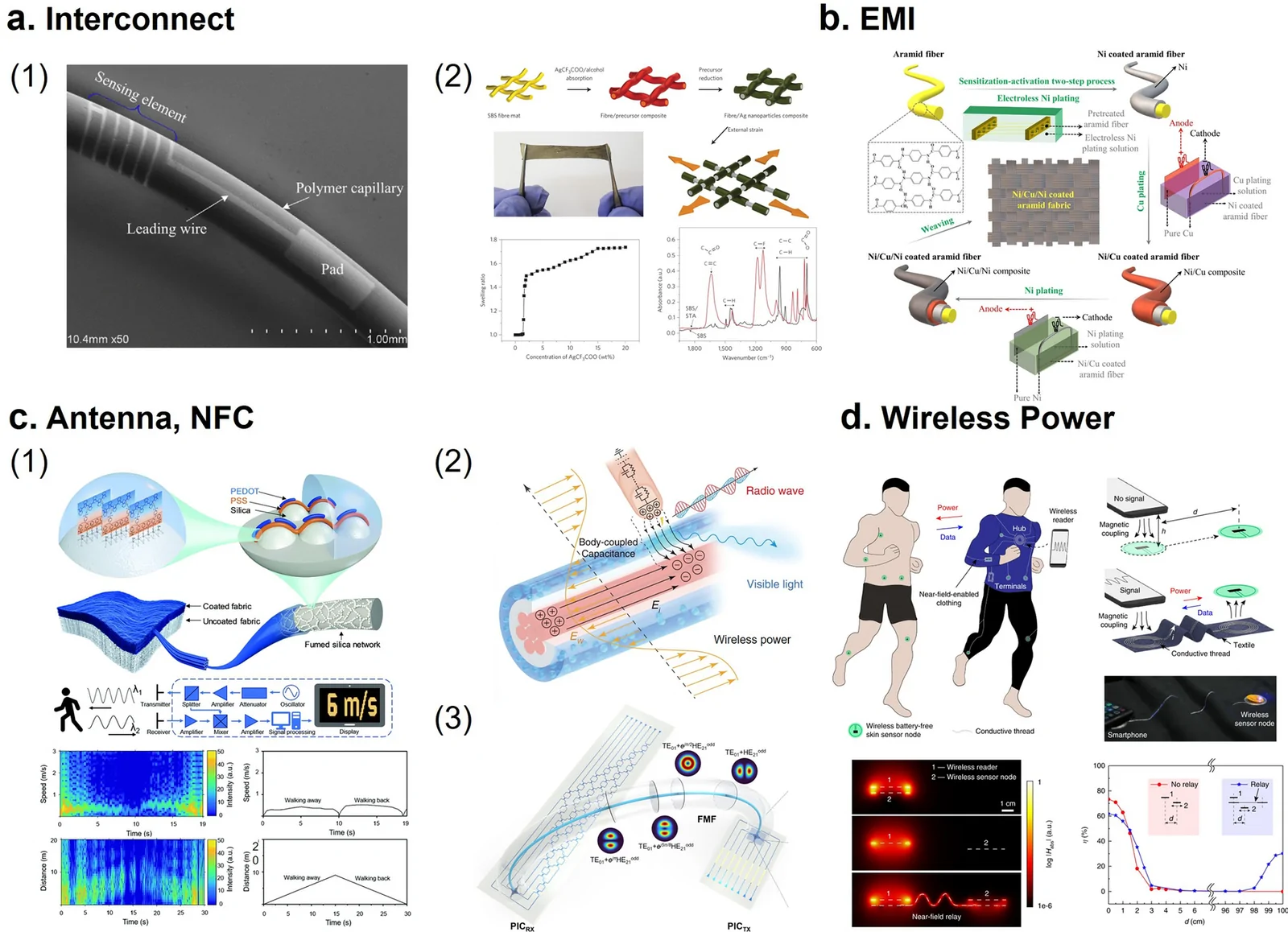
Communication Units on/in fiber. **a** Interconnect (1) SEM of fabricated flexible temperature sensor. Reprinted with permission from Ref. [56]. Copyright 2014, IEEE. (2) Fabrication and quantitative analysis of the composite non-woven mat of elastomer fibers and silver nanoparticles. Reprinted with permission from Ref. [218]. Copyright 2012, Springer Nature. **b** EMI: Fabrication process of AF@Ni/Cu/Ni by electroless Ni plating, Cu plating and Ni plating. Reprinted with permission from Ref. [226]. Copyright 2021, Elsevier. **c** Antenna (1) (top) Illustration of the microstructure of the PEDOT: PSS printed fabric. (bottom) RF signal transmission demonstration in a Doppler radar system. Reprinted with permission from Ref. [228]. Copyright 2020, Royal Society of Chemistry. (2) Design and principle of the body-coupled interactive fiber. Reprinted with permission from Ref. [229]. Copyright 2024, AAAS. (3) High-dimensional optical fiber communication system with reconfigurable integrated photonic processor. Reprinted with permission from Ref. [200]. Copyright 2024, Springer Nature. **d** Battery-free sensor nodes mounted on the skin and interconnected to a wireless reader through the near-field-enabled clothing. Reprinted with permission from Ref. [234]. Copyright 2020, Springer Nature

To enhance pattern resolution, Yang et al*.* developed the first programmable UV lithography system with alignment capabilities tailored for cylindrical substrates, enabling highly precise patterning on fiber-based geometries, as shown in Fig. 8a(1) [56]. This innovation supports the fabrication of flexible micro temperature sensors by easing exact temperature detection on deformable substrates.

High conductivity and stretchability are typically seen as mutually exclusive. However, Park et al. proposed a conductive composite mat composed of Ag nanoparticles and rubber fibers, enabling the development of highly stretchable circuits through a scalable and substrate-independent process suitable for large-area applications (Fig. 8a(2)) [218].

To ensure long-term durability and reliability of fiber-based electronic systems, effective encapsulation is essential for both washing resistance and electrical insulation. Encapsulation materials such as elastomers (e.g., PDMS, Ecoflex, and TPU), fluorinated polymers (e.g., PVDF, FEP), and ALD-derived oxides (e.g., Al_2_O_3_, SiO_2_) have been widely employed to form conformal protective layers around conductive or active fiber cores [219, 220]. These coatings prevent moisture penetration, and mechanical abrasion during repeated bending cycles. Multilayer encapsulation-combining a thin inorganic barrier layer for insulation with an outer flexible polymeric shell offers best balance between dielectric strength, flexibility, and breathability. For instance, Al_2_O_3_/elastomer bilayers and parylene-based coatings significantly enhance washing durability (> 100 cycles) while keeping electrical stability under deformation. Moreover, in situ dip-coating and plasma polymerization approaches enable scalability, and uniform encapsulation for complex fibers with no compensation in conductivity. Collectively, these encapsulation strategies are critical to achieve robust, washable, and electrically insulated fiber-integrated electronic systems suitable for real-world wearable applications.

### Wireless Communication Modules

This section discusses wireless communication modules vital for next-generation fiber-based electronic systems, building on the earlier focus on digital logic and signal processing. Traditional textile electronics rely on rigid silicon-based components, limiting integration with soft substrates and compromise the flexibility, energy efficiency, and user comfort. In contrast, integrating wireless communication directly into textiles allows for untethered operations, real-time data exchange, and intelligent system responses, crucial for the evolution of smart e-textiles.

High-performance processors require swift and reliable communication between storage and peripheral units to maximize computational power [221]. Conventional chipless systems, lacking integrated communication modules, constrain dynamic power routing, feedback control, and digital logic coordination. ICs with embedded wireless communication functions such as radio frequency (RF) transceivers, controllers, analog-to-digital converters (ADCs), and LCR meters address these limitations by enabling distributed data transmission, synchronization, and remote control within wearable platforms [222].

Incorporating wireless functionalities into fiber-based electronics ensures reliable data exchange and opens transformative possibilities for wearable systems. These include robust electromagnetic interference (EMI) shielding for signal fidelity, radio frequency (RF) antenna designs tailored for textile integration, and wireless power-delivery networks that help battery-free sensing and real-time edge processing. Collectively, these communication modules lay the groundwork for building fully autonomous, responsive, and scalable fiber-integrated electronic systems.

#### EMI Shielding for Signal Integrity

EMI shielding devices support signal integrity and ensure reliable operation in high-frequency environments within ICs. EMI from electronic medical equipment is a major source of signal distortion, leading to inaccurate measurement in bioelectronic systems [223].

The development of lightweight, flexible, and conformable wearable EMI shielding devices address these issues, making them ideal for next-generation electronics [202, 224]. High-quality EMI shielding fibers have been developed by modulating various pretreatment conditions, including refining, etching, and catalysis [225].

Tang et al. proposed a sandwich metal-structured conductive Aramid Fiber (AF) comprising amorphous Ni, crystalline Cu, and Ni as a promising EMI shield (Fig. 8b). This AF@Ni/Cu/Ni composite shows low resistivity, reduced density, and exceptional thermal stability, while supporting flexibility and mechanical integrity after 500 bending cycles. The multilayer interfaces of AF/Ni, Ni/Cu, and Cu/Ni enable multiple reflections and enhanced energy dissipation of incident electromagnetic (EM) waves, resulting in superior absorption and reduced reflection of EM radiation [226].

#### RF Antennas for Data Transmission

Wireless communication is a well-established method for data transmission in electronic systems that use emission and reception of EM waves. Unlike interconnects that route signals through physical media, antennas function as wireless transceivers, converting electrical signals into EM waves and vice versa. These waves consist of orthogonally oscillating electric (*E*) and magnetic (*B*) fields propagating at the speed of light (~ 3 × 10^8^ m s^−1^), enabling efficient data transmission without physical contact [227].

Recently, fiber-based antennas have gained attention for their compatibility with wearable platforms, aiming for thin, flexible, and washable designs that support RF performance under repeated stress. For instance, Li et al. developed a PEDOT-coated yarn antenna by controlling the crystallographic orientation and phase separation within a polymer matrix (Fig. 8c(1)). A fumed silica nanoparticle network guided PEDOT alignment on PET fabric, resulting in a Litz wire-like configuration with RF sheet resistance below 7 Ω [228]. The antenna proved a remarkable S11 value of − 50 dB and radiation efficiency of 28% at 2.35 GHz, supporting high-speed detection and real-time interaction in wearable applications such as blind aid.

Similarly, Yang et al. introduced an interactive fiber (*i*-fiber) capable of both wireless signal transmission and sensory feedback (Fig. 8c(2)). The fiber exhibited effective wireless transmission of electrical signals over a 30 m distance and retained optical signal power intensity of 10 nW cm^−2^ at 10 m [229]. Notably, the system enabled omnidirectional wireless transmission, delivering nearly uniform signal intensity in all directions, a feature critical for interactive and ambient smart textiles.

Lu et al. proposed a coaxial fiber antenna using a nano template-assisted PEDOT: PSS coating method (Fig. 8c(3)). This technique produces a conductive shell that minimizes skin effect losses, allowing for high RF conductivity [200]. The antenna proved a low return loss of 50 dB and radiation efficiency of 28% at 2.35 GHz, confirming its suitability for textile-integrated RF communication systems. For practical wireless signal links embedded in textile or fiber substrates, feedback control schemes are essential to support signal integrity under mechanical deformation or environmental changes. For example, the substrate may absorb moisture or be stretched, altering dielectric constant or tangential loss, thereby shifting the resonance or detuning the antenna (e.g., embroidered textile antennas show detuning and increased losses with moisture). Moreover, recent reviews on stretchable bioelectronics highlight the need for closed-loop feedback or adaptive modulation to compensate for changing coupling or signal path variations in deformable systems [230, 231]. Implementing small on-fiber/on-textile sensors or reference circuits (e.g., monitoring reflection, phase, or mismatch) can provide real-time feedback to tune matching networks, adjust transmission parameters, or adapt modulation to preserve data reliability.

#### Wireless Power and Edge Connectivity

Wireless power transfer (WPT) and edge connectivity are essential for next-generation wearable systems. These technologies drop bulky batteries and connectors for lightweight and flexible designs ideal for long-term use. In textile or fiber-integrated wireless power transfer (WPT) systems, mechanical strain (stretching, bending) and environmental factors (humidity, washing) can significantly change the electromagnetic coupling or dielectric properties of the textile medium, causing impedance mismatches and reduced power transfer efficiency. As reviewed in recent literature on wearable/bio-integrated flexible power/data systems, closed-loop feedback control (monitoring load voltage, reflected power, coupling coefficient, or phase of input currents) is crucial to dynamically adjust matching networks, tuning capacitors or compensation circuits [232, 233]. In practice, integrating small sensing elements or measurement across the fiber or textile (e.g., measuring current/voltage or reflection coefficients) allows adaptive re-tuning so that the receiver stays matched to transmitter despite mechanical or environmental perturbations. This helps support high efficiency and stable power delivery under washing, bending, or sweating conditions.

Inductive coupling for wireless charging is widely employed in consumer electronics like smartphones and has been explored for 3D IC integration and hinge-based data/power links. Lin et al. developed near-field-enabled clothing capable of proving wireless power and data connectivity between multiple points across the body (Fig. 8d). This system allows the deployment of battery-free sensor nodes that interconnect via proximity-based communication, forming a distributed body-area network embedded in functional textile patterns [234].

In addition, Guo et al. envisioned a hybrid fiber–wireless (FiWi) network architecture integrating centralized clouds and multi-access edge computing for smart textile platforms. This configuration enables real-time data analysis and reduces latency and dependency on external processors [235]. When paired with embedded microcontrollers and wireless modules (e.g., Bluetooth and Wi-Fi), these fabrics can autonomously collect, process, and send physiological or environmental data to cloud services or personal devices, expanding the scope of applications from healthcare to industrial monitoring. These advancements position wireless power transfer and edge connectivity as the cornerstones of next-generation e-textiles, enabling untethered operations, distributed sensing, and cloud-interfaced intelligence.

## Integration Strategies for Fully Functional Electronic Fibers

Conventional textile-based electronic systems often construct circuits by interlacing functional components along the warp and weft directions, with each fiber assigned a distinct function. These systems typically require the coating or integration of functional materials onto individual fibers [236]. However, the inherently rough, porous, and deformable nature of textile structures significantly hinders the formation of stable electrical contacts at fiber junctions, resulting in poor charge injections and transport characteristics. Consequently, fabric-junction-based systems often suffer from electrical instability and elevated contact resistance [237]. Moreover, woven electronic textiles show functional vulnerabilities under mechanical deformations, such as bending, stretching, and twisting. The non-uniform strain distribution across intersecting fibers can induce delamination, microcracks, or even permanent disconnection at junctions, leading to the degradation of electrical performance and long-term reliability. These deformation-induced issues present a critical challenge in realizing durable and high-performance electronic systems in dynamic or wearable environments [238, 239]. To overcome these limitations, researchers have focused on integrating multiple functional components directly into a single continuous fiber. This monolithic integration approach enhanced electrical reliability by reducing contact-related defects and enabling seamless internal connections. Compared with the traditional woven architecture, this method simplifies the overall structure and allows for more compact device designs. It also increases the integration density by refining the internal fiber layout and dropping the need for complex inter-fiber wiring [240].

Fiber-based electronic systems primarily use two types of interconnection strategies: serial and parallel [186, 241]. For example, connecting fiber-shaped supercapacitors in series increases the total voltage, while parallel arrangements reduce the internal resistance and allow each unit to run independently. The parallel approach is especially effective in keeping stable performance across systems under variable conditions.

Among the available techniques for integrating multiple components into fibers, thermal drawing is one of the most widely used and scalable. Since the original work of Fink et al. showed functional devices embedded within thermally drawn fibers, this approach has enabled the development of highly integrated and mechanically robust fiber systems. Recently, Loke et al. introduced a heterogeneous integration method that assembled discrete semiconductor chips onto a fiber platform [35]. This modular design supports the integration of advanced chips; however, the presence of rigid components may reduce comfort in wearable applications. To address this, flexible circuit substrates can be incorporated to improve softness and adaptability, although this often leads to a lower integration density and design complexity [242].

These strategies are a shift from distributed fabric-based circuits to more compact and integrated fiber systems. In the following sections, four representative approaches for implementing functional integration in electronic fibers are examined. These include discrete surface-mount assemblies, coaxial fiber designs, preform-based thermal drawing, and lithographic patterning techniques. Each offers distinct advantages and challenges, depending on the target functionality and use case.

### Surface-Mount and Discrete Assembly on Fiber Platforms

Another promising strategy for incorporating fiber-based electronics is the direct assembly of commercial devices onto fibers, which enables the proven performance of existing chips for ready use in textile structures. This integration is fully compatible with conventional semiconductor manufacturing processes, allowing for scalable high-throughput production and precision device packaging for enhanced protection and long-term durability. However, when 2D chips are embedded in woven textiles, their relatively rigid and flat geometries can cause discomfort and reduce the wearability of the fabric. Recent advances in chip miniaturization, reaching nanometer-scale dimensions, offer a potential solution by significantly mitigating wearability [243]. The continuous reduction in chip size, combined with further innovations in flexible packaging, is expected to enable the seamless integration of high-performance semiconductor devices into e-textiles without compromising softness, comfort, or conformability.

**Fig. 9:**
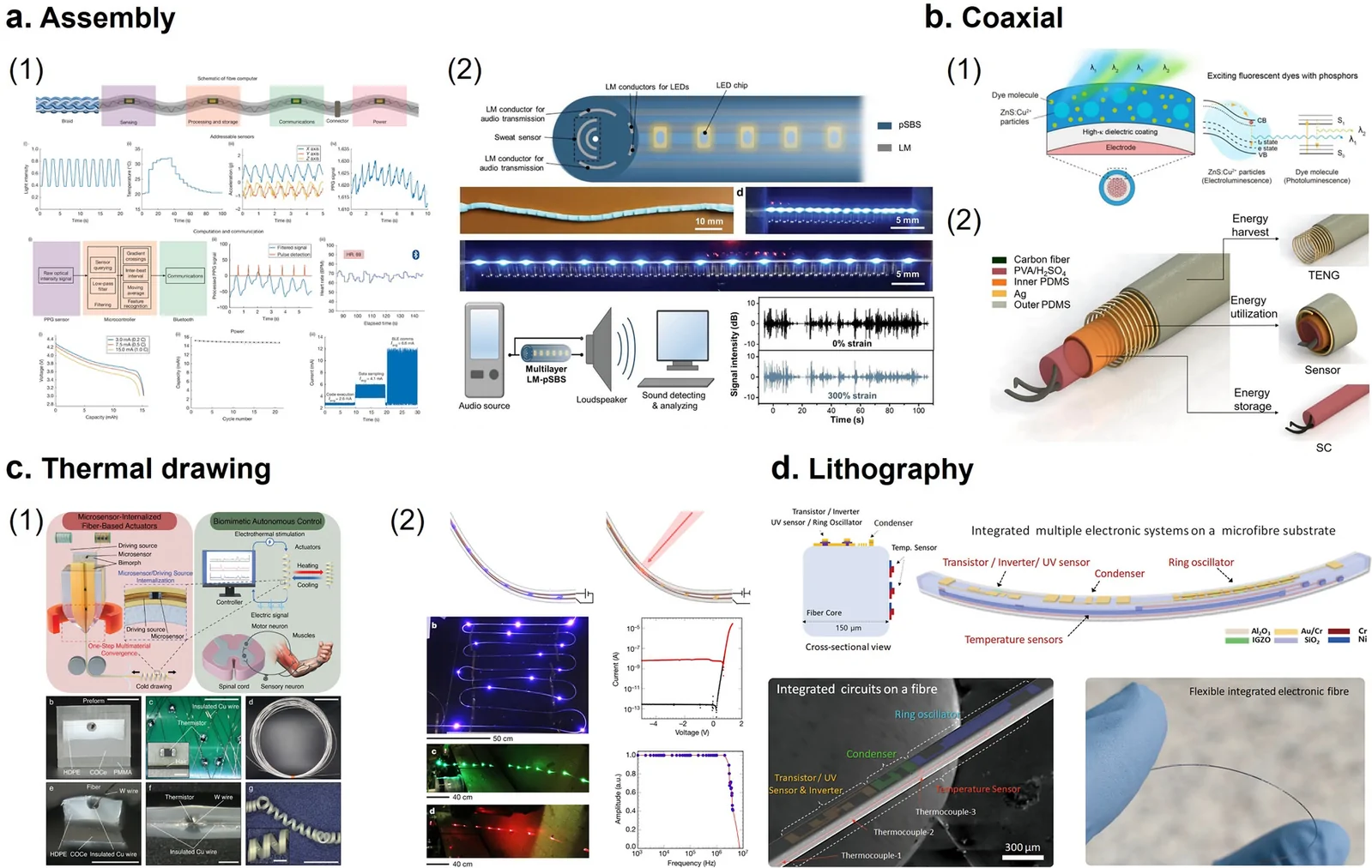
System on 1D substrate. **a** Assembly (1) Schematic of the fiber computer with distinct types of embedded devices, including sensors, MCUs for processing and storage, and communication devices, connected by four electrodes to form a linear I2C bus. Reprinted with permission from Ref. [244]. Copyright 2025, Springer Nature. (2) Schematic illustration showing the structure of a permeable, high-integration-density, and multifunctional LM-pSBS fiber incorporating three layers of LM circuits. Reprinted with permission from Ref. [245]. Copyright 2023, Wiley–VCH GmbH. **b** Coaxial (1) Electroluminescence excited fluorescent dye strategies to construct multicolor *i*-fibers. Reprinted with permission from Ref. [229]. Copyright 2024, AAAS. (2) Schematic structure diagrams of the energy fiber for energy harvesting, storage, and use (including TENG, sensor, and SC). Reprinted with permission from Ref. [248]. Copyright 2021, American Chemical Society. **c** Thermal drawing (1) Scheme and fabrication process of fiber-based actuators. Reprinted with permission from Ref. [250]. Copyright 2024, Wiley–VCH GmbH. (2) Light-emitting and high-bandwidth photo-detecting fibers. Reprinted with permission from Ref. [41]. Copyright 2018, Springer Nature.** d** Lithography: Cross-sectional and three-dimensional scheme of the device elements fabricated on the microfiber substrate. Reprinted with permission from Ref. [240]. Copyright 2022, Springer Nature

Gupta et al. developed a monofilament-based distributed-inference computing system by transforming a conventional 2D pad layout into a 3D fiber configuration using a foldable interposer (Fig. 9a(1)). This approach enables integrating various components, including a microcontroller, sensors (accelerometer, temperature, light, and PPG), communication module (BLE), and LED within a compact millimeter-scale fiber structure [244]. The devices are interconnected via an Inter-Integrated Circuit (I2C) bus, allowing efficient data processing and transmission while minimizing power consumption. Furthermore, the system showed outstanding mechanical compliance, with stretchability exceeding 60%.

Li et al*.* proposed a strategy for stencil printing of Liquid Metal patterns on porous poly(styrene-block-butadiene-block-styrene) (pSBS) fiber surface for multilayer electrical circuits, enabling the fabrication of multifunctional fibers with distinct layers dedicated to stretchable lighting, data transmission, and biochemical sensing (Fig. 9a(2)). The fiber incorporates parallel LM conductors on the outer layer to power the embedded LEDs, LM conductors on the middle layer to send audio signals, and a capacitive inner layer with parallel LM electrodes for sweat sensing [245]. Even under 300% strain, the printed LM conductors and LEDs remained fully functional. Surface treatment with (3-Aminopropyl) triethoxysilane (APTES) made fiber hydrophilic, allowing the inner-layer capacitor to reliably detect both volume and NaCl concentration in artificial sweat under stretched and relaxed conditions. Furthermore, by using its multimodal sensing capability, the LM-pSBS fiber enables an integrated artificial neuron to simultaneously check temperature and pressure variations and send electrical signals, showing its potential for use in wearable neuromorphic systems.

### Coaxial and Core–Shell Fiber Architectures

Coaxial configuration is beneficial for integrating systems into single fibers by using a core–shell design that enables rapid charge transfer by integrating electrodes as the core and functional layers as the shell [246]. This configuration is particularly helpful for fiber PDs and transistors and can be fabricated using various methods, such as dip-coating, coaxial spinning, and 3D printing [15]. The coaxial structure enables the seamless integration of diverse materials and device architectures, making it highly suitable for a wide range of applications. However, keeping a stable interface between the substrate electrode and the active material on a highly curved fiber surface stays a significant challenge [247].

Yang et al. developed a soft, thin fiber that can perform wireless transfer and sensory processing and shows various forms of feedback (Fig. 9b(1)). The interactive fiber (*i*-fiber) consisted of three functional layers: an antenna core (silver-plated nylon fibers) for inducing an alternating electromagnetic (EM) field, a dielectric layer (BaTiO_3_ mixed resin) for storing coupled EM energy, and an optical layer (ZnS: Cu^2+^ mixed resin) for visualizing the electric field [229]. Each layer was fabricated using a layer-by-layer coating approach using equipment and protocols aligned with industry standards. When a soft *i*-fiber is placed on the palm, the interface contact capacitance effectively captures the ambient electromagnetic energy, thereby enabling the *i*-fiber to emit light. Furthermore, simultaneous generations of optical and electrical signals were shown upon finger contact with an embroidered *i*-fiber. The fiber can perform wireless transfer, sensory processing, and feedback, making it a building block for electronic textiles. By introducing a previously unexplored body-coupling ambient EM energy harvesting strategy, this study suggests the potential for chip-free wireless interactions in future textile-based optical communication systems.

Han et al. developed a multifunctional coaxial energy fiber based on a fiber-shaped TENG, Supercapacitor, and pressure sensor in a coaxial design (Fig. 9b(2)). The fibrous TENG delivers a peak power output of 2.5 µW and can drive small devices such as an electronic watch and temperature sensor [248]. Additionally, the integrated pressure sensor shows high sensitivity (1.003 V kPa^−1^ below 23 kPa), enabling real-time finger motion detection and serving as a tactile interface for applications like a fibrous electronic piano.

### Preform-Based Thermal Drawing of Multicomponent Fibers

Thermal drawing enables precise placement of functional components within a fiber preform before the fiber is thinned. This approach is particularly helpful for applications that require isolation between signals and protection of embedded devices. It supports the integration of multiple functionalities in a space-efficient manner by embedding electrical, optical, or chemical modules within a limited fiber cross section. Additionally, this method preserves exact timing performance across all integrated functions, while allowing the use of soft polymers and composite materials that enhance flexibility. This process inherently seals the fiber structure, offering effective protection against environmental exposure, thereby improving the durability and reliability of fiber-based systems in wearable and biomedical applications.

Kim et al. introduced a multifunctional neural probe fabricated through microwire thermal drawing, designed for dual-mode synaptic recording and stimulation in a rodent brain model [249]. Lee et al. developed a microscale fiber actuator that autonomously controlled its motion by integrating a heating element and temperature sensor into a single-fiber strand (Fig. 9c(1)). This configuration enables real-time sensing and closed-loop feedback control using a proportional-integral-derivative scheme [250]. The actuator achieved stable and repeatable movements, allowing it to perform complex tasks such as finger bending, object grasping, and transport, proving its potential in soft robotics and wearable actuation technologies.

Rein et al*.* proved the integration of *p*–*i*–*n* diodes and light-emitting devices into a single-fiber structure by carefully aligning conductive wires with semiconductor elements during the thermal drawing process (Fig. 9c(2)). This technique enables the construction of hundreds of light sources and PDs in parallel within a single fiber [41]. Optical communication between the fibers was successfully shown through a bidirectional link working at 3 megahertz. The same system was also capable of monitoring heart rate, suggesting a strong potential for fabricating photonic sensors used in wearable health monitoring.

### Direct Semiconductor Device Integration on 1D Fiber Substrate

The direct integration of semiconductor devices onto 1D fiber substrates presents a transformative approach for constructing high-density multifunctional electronic systems with inherent mechanical flexibility. Unlike traditional planar platforms, fiber substrates present fabrication challenges owing to their curved geometry, limited surface area, and dynamic deformation during use. Nevertheless, advances in high-resolution lithographic techniques have enabled the direct fabrication of semiconductor devices on cylindrical fibers with increasing precision, thereby enabling scalable and compact system integration for next-generation e-textiles. Lithography stays the foundational method for microfabrication, enabling the controlled transfer of intricate circuit patterns onto a substrate. When tailored for fiber applications, lithographic techniques can achieve micro-scale resolutions on curved surfaces, supporting the realization of dense circuit layouts and multicomponent integration. Furthermore, their compatibility with reel-to-reel processing offers a route for scalable production of fiber-based devices with reproducible performance characteristics [251].

In a representative study, Hwang et al*.* proved monolithic SoF by embedding various semiconductor devices directly onto the four sides of a square-shaped microfiber through sequential thin-film deposition and lithographic patterning (Fig. 9d). This architecture enabled the fabrication of a fully functional microprocessor within a 19.3 cm fiber segment, integrating logic units, sensing elements, and data-processing capabilities in a single structure [240]. The square geometry of the fiber substrate was key to maximizing integration density and functional diversity. Extending this concept, Markiewicz et al*.* introduced a lithography-based platform that incorporated both photonic and microfluidic elements within a unified fiber-compatible structure [252]. This integration allows seamless interfacing between the external optical and fluidic systems, thereby offering new possibilities for wearable biochemical sensing, optical communication, and lab-on-fiber applications.

While high-precision lithography remains the dominant method for defining device geometries on fiber substrates, recent progress in alternative patterning and transfer-based technologies offers complementary routes toward scalable, flexible, and low-cost integration. Mask-assisted photolithography and soft lithography using elastomeric stamps enable the replication of micro/nanoscale patterns onto curved or deformable fiber surfaces with high fidelity, providing simplistic and adaptable alternative to conventional systems [253]. Microcontact printing and inkjet printing have appeared as powerful additive methods for depositing conductive inks, semiconductors, and dielectric materials directly onto fibers, minimizing material waste and allowing pattern reconfiguration without complex vacuum processes. These printing-based methods are compatible with roll-to-roll and reel-to-reel manufacturing, offering strong industrial scalability for continuous fiber functionalization [254]. Moreover, transfer printing techniques including dry transfer, lamination-assisted transfer, and specific pick-and-place enable the integration of prefabricated micro/nano devices (such as thin-film transistors, sensors, and photonic chips) onto fiber substrates with micro-level alignment precision. This strategy circumvents thermal and chemical compatibility issues inherent to direct fabrication, easing heterogeneous integration of inorganic or 2D materials within soft fiber matrices [255, 256]. Collectively, these emerging patterning and transfer technologies complement lithographic approaches by expanding the freedom of design, scalability, and material versatility needed to realize fully integrated, multifunctional SoF platforms.

### Multi-Device Integration on Fiber Substrates

The multifunctional SoF platforms demand seamless integration of multiple active and passive components such as transistors, diodes, memory elements, sensors, and energy modules within a single continuous fiber. Fiber architecture presents unique challenges arising from their cylindrical geometry, limited surface area, and non-planar strain distribution, complicated alignment, electrical isolation, and interconnect routing. Recent progress has shown that coaxial and multi-core fiber geometries offer promising solutions by providing spatial separation of conductive and insulating domains, thereby enabling parallel stacking and vertical signal routing without compromising flexibility [257]. For example, thermally drawn multi-material fibers and performance-based architectures have been used to embed field-effect transistors, photodetectors, and micro-supercapacitors within a single cross-section, proving stable operation under bending and twisting.

Hybrid integration strategies enhance functionality by combining top-down microfabrication (e.g., lithography, sputtering, ALD) with bottom-up assembly methods (e.g., coating, electrospinning, and printing) to construct heterogeneous device layers on curved substrates. This hybrid approach allows each functional module to be independently refined and interconnected through conductive substrates, liquid–metal pathways, or flexible printed connectors. In addition, transfer printing and lamination-assisted assembly enable deterministic placement of prefabricated chips or thin-film devices onto fiber surfaces with sub-micron precision, expanding the range of materials beyond polymeric systems to include 2D semiconductors, metal oxides, and perovskite layers [240, 258]. However, the increasing device density along the fiber introduces new challenges in thermal management, electromagnetic interference, and mechanical strain coupling, needing advanced encapsulation and adaptive signal compensation.

Upon futuristic, AI-assisted design tools and digital-twin simulations will play a pivotal role in refining multi-device integration, predicting strain-induced electrical adaptations, and automated alignment during fabrication [259]. The combination of coaxial multi-core fiber architectures, modular device stacking, and intelligent control algorithms will ultimately enable compact, multifunctional SoF platforms that integrate sensing, computing, memory, and wireless communication within a single-fiber strand paving the way toward autonomous, and self-adaptive textile systems.

## Future Perspectives and Outlooks

Over the past two decades, fiber-based electronics have grown substantially, propelled by advances in materials science, device miniaturization, and scalable integration techniques. These developments enable system-level functionalities, ranging from basic sensing and communication to neuromorphic computing and embedded processing within fiber architectures. Section 4 highlights multiple studies, proving the viability of embedding diverse electronic components into a single-fiber substrate, achieving performances comparable to conventional planar systems [17]. The integration of sensing, data processing, memory, actuation, and wireless communication within the fibrous form factor highlights the transformative potential of system-on-fiber electronics.

However, several critical challenges must be addressed before fiber-integrated systems can transition from research prototypes to real-world deployments. These challenges include developing encapsulation strategies, scalable manufacturing platforms, standardized performance evaluation metrics, and seamless integration of multifunctional modules. Addressing these challenges is essential to fully realize the potential of intelligent fiber electronics.

### Material Innovations and Robust Encapsulation

Progress in fiber-based electronics relies heavily on material innovation for multifunctionality, reliability, and adaptability. Incorporating nanomaterials, conductive polymers, and hybrid fillers into fiber substrates enhances electrical conductivity and mechanical compliance [260, 261]. Nanoscale optimization, such as the development of nanoporous or hierarchical architecture, improves electrochemical performance by increasing surface area and easing ion and charge transport [262, 263]. Thermal and mechanical robustness is essential for long-term stability under repeated bending, laundering, and exposure to environmental stressors. Carbon-based thermal interface materials and corrosion-resistant metals show promise for thermal management and structural reinforcement [264]. To simplify fabrication, multifunctional materials that fulfill electrical, mechanical, and environmental roles are helpful.

Device encapsulation is crucial for ensuring the mechanical durability and environmental resistance of fiber-based systems. Encapsulation layers must prevent abrasion-induced failures while staying comfortable and skin compatible. Biocompatibility and nontoxicity are essential for wearable systems in prolonged skin contact. Although encapsulation, interconnect design, and scalable manufacturing have improved the durability of fiber electronic systems, current strategies stay inadequate for long-term, scalability applications. Multilayer polymer encapsulants combining elastomers (PDMS, TPU) with inorganic ALD coatings (Al_2_O_3_, SiO_2_) offer preliminary protection but suffer from delamination, micro-cracking, and moisture ingress after extended bending or washing, retaining < 70% electrical stability beyond 1,000 cycles. Similarly, conductive yarn and stitched interconnects (Ag-coated nylon, CNT, or liquid–metal composites) improve flexibility but show contact resistance drift > 20% due to junction fatigue and poor adhesion at fiber interfaces. Encapsulation of these joints adds stiffness, compromising textile comfort and scalability. On the manufacturing side, roll-to-roll coating, weaving, and 3D knitting enable meter-scale processing but face > 10% yield loss from non-homogeneous coating and fiber misalignment, intensifying the real-time defect detection and process feedback. Collectively, these limitations underscore the need for adaptive self-healing encapsulants, coaxial fiber interconnects, and AI-driven closed-loop manufacturing to achieve reliable, washable, and industrially scalable SoF architectures [151, 265, 266].

Recent advances in AI and machine learning (ML) have introduced transformative approaches for refining both materials and device architectures in SoF technologies. By proving data-driven correlations among processing parameters, structural features, and performance outputs, AI frameworks enable rapid exploration of high-dimensional design spaces that are otherwise inaccessible through empirical experimentation. Bayesian optimization, genetic algorithms, and reinforcement learning have been employed to show polymer compositions, conductive ink formulations, and dielectric stacks that jointly maximize electrical conductivity, stretchability, and wash durability under textile-relevant deformation. At the device level, surrogate and physics-informed neural network models can predict and optimize characteristics such as threshold voltage, switching energy, retention, and bias stability for OECTs, memristors, and neuromorphic fibers. Closed-loop autonomous experimentation systems, integrated through in-situ characterization with active learning, emerge as a powerful paradigm for real-time feedback control of fabricating conditions enabling self-correcting pipelines. In addition, AI-assisted electromagnetic simulators and differentiable design tools allow inverse optimization of antenna geometries and wireless power modules, claiming impedance matching under mechanical strain and humidity variations typical of textile substrates [267]. These developments collectively mark a shift toward self-optimizing, adaptive manufacturing, where AI-driven frameworks accelerate discovery, reduce experimental cycles, and guide scalable industrial translation of multifunctional SoF platforms. Future research should focus on standardized mechanical–electrical durability testing and AI-driven parameter optimization to unify evaluation protocols across material systems. In addition, the table below summarizes the material properties with their key parameters and limitations Table 3.

**Table 3:** A comprehensive review of material property, key parameters and limitations

Materials	Electrical Conductivity (S m^−1^)	Rate of Resistance change (ΔR/R0)	Endurance	Key Features/Limitations	Refs
EGaIn (Liquid Metal)	3.4 × 10^6^ – 6.3 × 10^6^	< 5%	1000 cycles @50% strain	High conductivity, self-healing, oxidation-sensitive	[268]
Galinstan (LM alloy)	3.5 × 10^6^	< 10%	2,000 cycles @100% strain	Excellent ductility, require encapsulation	[269]
Ag Nanowire Network	1 × 10^5^ – 5 × 10^5^	10%–20%	1,000 cycles @30% strain	Good conductivity, moderate fatigue resistance	[270]
CNT–Elastomer Composite	1 × 10^3^ – 1 × 10^4^	20%–30%	1,000 cycles @50% strain	Excellent flexibility, less conductivity	[271]
PEDOT: PSS fiber	1 × 10^2^ – 1 × 10^3^	40%–60%	500 cycles @30% strain	High flexibility, moisture-sensitive	[272]
Conductive Hydrogel	10 – 10^3^	50%–70%	500 cycles @100% strain	Soft, biocompatible, poor stability	[273]
rGO	–	<1%	10,000 cycles @30% strain	Human monitoring, complex robotic movements	[114]
CVD- graphene	–	–	–	Haptic touching and slightmovements	[274]
Scaffold PU fiber	10	<20%	280 cycles @15% strain	Strain feasibility	[275]
Graphene	0.15	10%	1000 cycles @50% strain	Motion and health monitoring	[276]
Silicone	0.1 × 10^3^ – 1 × 10^3^	<5%	10,000 cycles @30% strain	Durability and highly dielectric	[277]
CNTs	–	0.13%	–	Flexible and thermally unstable	[278]
Graphene nanoplatelets	150	–	–	Easily bendable flexographic	[279]
PEDOT: PMMA	–	600	244 cycles @70% strain	Intense polymerization and deformable	[280]
Organic materials	–	1%	–	Easily oxidizable	[281]

### Advanced Manufacturing for Complex Fiber Systems

Fabricating electronics differs from conventional lithographic processes, as fibers require precise deposition and alignment on curved deformable substrates. This calls for scalable fiber-compatible manufacturing methods compatible with textiles while supporting high precision. Accurate control of interfacial properties is especially important for multilayer architectures. For example, the interface between organic semiconductors and dielectric layers significantly influences the charge transport in OFETs [282]. Advanced deposition techniques and interface engineering strategies are being explored to enhance the electrical and mechanical performances of fiber-integrated systems. Additionally, large-scale fabrication must ensure reproducibility, device uniformity, and compatibility with post-processing, such as weaving and knitting, to ease commercialization.

### Testing and Validation of Flexible Fiber Circuits

To rigorously evaluate fiber-based ICs alongside silicon-based ones, standardized testing protocols are essential. Conventional IC testing encompasses electrical parameter monitoring, functional and reliability assessments, and package-level evaluations [283]. However, these methods are tailored for rigid planar substrates and do not apply to fibrous systems. Fiber-based electronics must withstand mechanical deformations such as bending, twisting, and stretching during regular use, while conventional tensile testing does not replicate these dynamic conditions. Furthermore, the lack of standardized mechanical and electrical evaluation protocols hinders comparability across studies and stifles technological progress [284]. Therefore, developing fiber-specific evaluation methodologies that assess both electrical performance and mechanical durability is critical for industrial implementation.

### Toward Multifunctional and Intelligent Optical Fiber Platform

Recent progress in fiber electronic systems has proven prototypes for wearable health monitoring yet transitioning it into quantitatively verifiable system-level performance remains a challenge. In representative configurations, fiber-based strain and pressure sensors achieve sensitivities of 0.2–1.5 kPa^−1^, with response times below 50 ms and reliable operation under > 10,000 bending cycles, making them suitable for motion detection and pulse monitoring. Similarly, fiber bioelectrodes exhibit skin-contact impedance below 20 kΩ cm^2^ and support stable biopotential acquisition (SNR > 30 dB) during dynamic movement [285]. For physiological monitoring, fiber-integrated electrochemical sensors have shown detection limits down to 10^–7^–10^–9^ M for biomarkers such as glucose, lactate, or cortisol, and support signal drift < 5% after 100 wash cycles, confirming their environmental robustness [286, 287].

At the system scale, woven fiber arrays have achieved spatial resolution of ~ 2 mm, wireless data transmission rates up to 1 Mbps, and operational lifetimes exceeding 10^4^ mechanical cycles under 30% strain. Integrated power modules, including fiber supercapacitors and photovoltaic fibers, deliver energy densities of 5–10 mWh cm^−2^ and output stability > 95% after cyclic bending [288]. These quantitative indicators prove that SoF systems are approaching the performance thresholds required for real-time, continuous, and washable wearable monitoring. Future research should focus on developing standardized testing protocols and AI-assisted reliability modeling to quantitatively correlate device-level performance with overall system stability, ensuring reproducibility and regulatory readiness for biomedical applications [261].

**Fig. 10:**
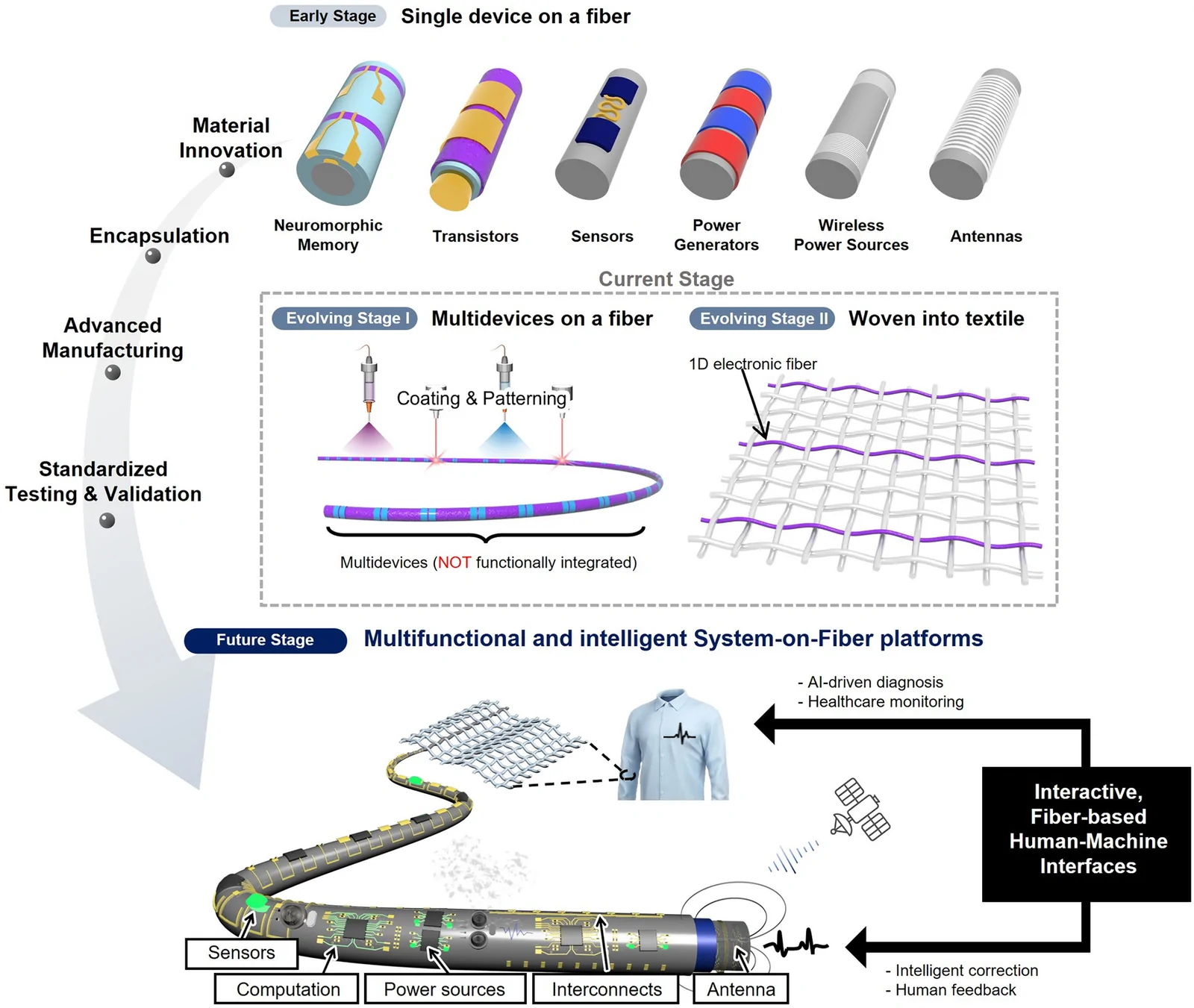
Evolution roadmap of System-on-Fiber (SoF) technologies toward multifunctional and intelligent fiber platforms. The illustration depicts the progressive transition from early-stage, single-device fibers to the current evolving stage, where coating, patterning, and weaving strategies enable the integration of multiple devices on a single fiber or within a textile. The envisioned future stage involves fully integrated SoF architectures that embed sensing, computation, power management, interconnects, and wireless communication modules within a single continuous fiber. Such physical and functional integration of devices ultimately leads to interactive human–machine interfaces seamlessly embedded in textiles

To progress beyond single-function devices, future progress in SoF technology requires a well-defined and quantitatively guided roadmap that integrates advances across materials science, photonics, electronics, and AI. The next generation of SoF systems will hinge on proving a cohesive architecture that couples optical, electronic, and intelligent design layers within a single continuous fiber platform as illustrated in Fig. 10. Initial priority is improving material durability and environmental stability. Current encapsulation layers typically elastomeric polymers or thin-film oxides suffer from moisture permeation, cracking, and delamination under repeated bending or laundering. Practical solutions include hybrid polymer–oxide coatings, self-healing elastomers, and strain-decoupled multilayer barriers. Identifying optimal material combinations will benefit from AI-assisted material screening, which can predict degradation pathways and guide selections compatible with thermal drawing, ALD, or printing [289].

In the mid-term, reducing the manufacturing process complexity through scalable, textile-compatible fabrication workflows. Thermal drawing, ALD, inkjet printing, and spinning currently operate as disjointed steps, leading to yield loss and variability. Convergence toward hybrid roll-to-roll and drawing processes, supplemented with digital-twin based process monitoring and in-line machine-vision inspection, can significantly improve reproducibility and lower production costs. Modular design of plug-and-play fiber building blocks including standardized connectors, embedded interconnects, and packaged device modules will further streamline assembly into woven or knitted systems [290]. At this stage, AI-assisted circuit design and reinforcement-learning-based feedback control will enable adaptive impedance matching and real-time signal reconfiguration under mechanical strain or environmental fluctuations [291].

As for the long term, SoF platforms address system-level challenges, including high-density interconnect reliability, signal integrity during deformation, and cross-device coordination. Coaxial architecture, conductive-core fibers, and self-healing liquid–metal pathways offer promising directions but require standardized testing for fatigue (> 10^4^ cycles), washability (ISO 6330), and stretch endurance (ASTM D4964). Establishing such benchmark criteria is essential for comparing fabrication methods and certifying consumer-grade products [292]. Achieving these milestones will require interdisciplinary collaboration across materials informatics, AI-driven design automation, optoelectronic integration, and large-scale manufacturing engineering. Collectively, industrial translation will demand a clear cost-control strategy. Transitioning from laboratory equipment (vacuum sputtering, slow ALD cycles, multi-step photolithography) toward solution-processable dielectrics, spatial ALD, low-temperature metallization, and textile-integrated printing systems will reduce processing time and equipment overhead. Collaborative frameworks between academia, textile manufacturers, and electronics industries are needed to define regulatory pathways, recyclability standards, and supply-chain integration.

The integration of multimodal capabilities such as sensing, computing, and actuation into a single fiber presents a significant challenge. A promising approach is to use multifunctional materials that can both sense stimuli and function as memory elements, reducing processing steps and minimizing the interconnect complexity [293]. Additionally, integrating advanced sensing modalities like radar and LiDAR, can enable textiles to support context-aware applications, including robotics, assistive technologies, and health monitoring [294, 295]. Overall, coordinated advances in materials, manufacturing, interconnect engineering, and standardization, SoF technologies can transition from laboratory prototypes to scalable, robust, and intelligent electronic textiles ready for real-world applications.

### Outlook on Standardization and Industrial Translation

From SoF technologies to advanced laboratory-scale prototypes and real-world commercialization, a coordinated roadmap encompassing standardization, scalable manufacturing, and industrial transition is essential.

(i) Developing universal testing and certification standards to ensure performance consistency over research and industry defining metrics for electrical retention (> 90% after 50 wash cycles), mechanical endurance (> 10^4^ bending cycles), and biocompatibility following ISO and ASTM protocols. Parallel efforts focus on process standardization, where scalable techniques such as roll-to-roll thermal drawing, spatial ALD, and automated fiber weaving are customized for reproducibility, cost efficiency, and integration into textile production.
(ii) Design modularity for the development of standardized fiber connectors, encapsulants, and interconnects that can work seamlessly with garment assembly and consumer electronics infrastructure.
(iii) Deployment of AI-driven digital twins and machine-vision quality inspection will enable closed-loop control, ensuring yield and analytical defect alteration on high-volume manufacturing.
(iv) Industrial collaboration frameworks involving academia, textile manufacturers, electronics companies, and regulatory agencies must be proven to define certification pathways, safety testing, and recycling guidelines for wearable electronic textiles.

These coordinated steps from material qualification to automated production and regulatory alignment are decisive in transforming SoF systems from niche laboratory demonstrations into durable, standardized, and smart textiles for healthcare, sports, defense, and wearable applications [296].

## Abbreviations

SoF: System-on-Fiber
5G: Fifth-generation
AI: Artificial Intelligence
IoT: Internet-of-Things
VLSI: Very Large Scale Integration
SoC: System-on-Chip
MEMS: Microelectromechanical systems
3D: Three-dimensional
ICs: Integrated Circuits
HBM: High bandwidth memory
1D: One-dimensional
2D: Two-dimensional
CVD: Chemical Vapor Deposition
WOLED: White organic Light emitting diode
Al_2_O_3_: Aluminum oxide
Au: Gold
Ag: Silver
CTAC: Capillary Tube-Assisted casting
PVDF: Poly(vinylidene fluoride)
TrFE: Trifluoro ethylene
*T*_g_: Glass transition temperature
PVD: Physical vapor deposition
ALD: Atomic layer deposition
μm: Micrometer
PEDOT: Poly(3,4-ethylenedioxythiophene)
PSS: Polystyrene sulfonate
FETs: Field effect transistors
OECTs: Organic electrochemical transistors
BBL: Poly(benzimidazo benzophenanthroline)
CPU: Central Processing Unit
RAM: Read-Access memory
NAND: Universal logic gate
ECD: Electrochemical deposition
nJ: Nano Joules
fJ: Femto Joules
ns: Nanoseconds
ANNs: Artificial Neural Networks
STP: Short-term plasticity
LTP: Long-term plasticity
CMOS: Complementary metal-oxide Semiconductor
LEDs: Light emitting diodes
AC: Alternative current
DC: Direct current
ESD: Electrostatic discharges
FWHM: Full-width half-maximum
LiFi: Light-fidelity
fOED: Fibriform organic electrochemical diode
CuI: Copper iodide
ms: Millisecond
I-V: Current versus voltage
PVA: Polyvinyl alcohol
PDs: Photodetectors
nW: Nano Watt
Hz: Hertz
ppm: Parts per million
ppb: Parts per billion
ECG: Electrocardiogram
e-textiles: Electronic textiles
CTFs: Conductive tough fibers
IPX8: Ingress protection
MPa: Mega Pascals
kS: KiloSiemens
cm: Centimeter
dB: Decibel
NOR: NOT OR logic gate
Zn: Zinc
PVK: Polyvinyl carbazole
ZnO: Zinc oxide
PMMA: Poly(methyl methacrylate)
CNT: Carbon nanotube
SPF: Structured polymer fiber
Cu: Copper
LM: Liquid metals
RF: Radio-frequency
ADCs: Analog-to-digital converters
LCR meter: Inductance, Capacitance and Resistance meter
EMI: Electromagnetic interference
AF: Aramid Fiber
Ni: Nickel
EM: Electromagnetic
PET: Poly(ethylene terephthalate)
*i*-fiber: Interactive fiber
WPT: Wireless power transfer
FiWi: Fiber Wireless
WiFi: Wireless network
PSBS: Poly(styrene-butadiene-styrene)
APTES: (3-Aminopropyl) triethoxysilane
NaCl: Sodium chloride
I2C: Inter-Integrated Circuit
BaTiO_3_: Barium Titanate
ZnS: Zinc sulfide
Cu^2+^: Copper ions
TENG: Triboelectric Nanogenerators
*p*-*i*-*n* diodes: *P*-Type, intrinsic, *n*-type diodes
MCUs: Microcontrollers
LiDAR: Light detection and ranging
